# Lenvatinib improves anti-PD-1 therapeutic efficacy by promoting vascular normalization via the NRP-1-PDGFRβ complex in hepatocellular carcinoma

**DOI:** 10.3389/fimmu.2023.1212577

**Published:** 2023-07-21

**Authors:** Jieying Yang, Zhixing Guo, Mengjia Song, Qiuzhong Pan, Jingjing Zhao, Yue Huang, Yulong Han, Dijun Ouyang, Chaopin Yang, Hao Chen, Muping Di, Yan Tang, Qian Zhu, Qijing Wang, Yongqiang Li, Jia He, Desheng Weng, Tong Xiang, JianChuan Xia

**Affiliations:** ^1^ Department of Biotherapy, Sun Yat-sen University Cancer Center, Guangzhou, China; ^2^ Collaborative Innovation Center for Cancer Medicine, State Key Laboratory of Oncology in South China, Sun Yat-sen University Cancer Center, Guangzhou, China; ^3^ Department of Ultrasound, Sun Yat-sen University Cancer Center, Guangzhou, Guangdong, China; ^4^ Department of Oncology and Translational Medicine Center, The Second Affiliated Hospital, Guangzhou Medical University, Guangzhou, China; ^5^ Guangzhou Laboratory, Guangzhou, China; ^6^ Intensive Care Unit, Sun Yat-sen University Cancer Center, Guangzhou, Guangdong, China

**Keywords:** lenvatinib, vascular normalization, immune infiltration, anti-PD-1, hepatocellular carcinoma

## Abstract

**Introduction:**

The limited response to immune checkpoint blockades (ICBs) in patients with hepatocellular carcinoma (HCC) highlights the urgent need for broadening the scope of current immunotherapy approaches. Lenvatinib has been shown a potential synergistic effect with ICBs. This study investigated the optimal method for combining these two therapeutic agents and the underlying mechanisms.

**Methods:**

The effect of lenvatinib at three different doses on promoting tissue perfusion and vascular normalization was evaluated in both immunodeficient and immunocompetent mouse models. The underlying mechanisms were investigated by analyzing the vascular morphology of endothelial cells and pericytes. The enhanced immune infiltration of optimal-dose lenvatinib and its synergistic effect of lenvatinib and anti-PD-1 antibody was further evaluated by flow cytometry and immunofluorescence imaging.

**Results:**

There was an optimal dose that superiorly normalized tumor vasculature and increased immune cell infiltration in both immunodeficient and immunocompetent mouse models. An adequate concentration of lenvatinib strengthened the integrity of human umbilical vein endothelial cells by inducing the formation of the NRP-1-PDGFRβ complex and activating the Crkl-C3G-Rap1 signaling pathway in endothelial cells. Additionally, it promoted the interaction between endothelial cells and pericytes by inducing tyrosine-phosphorylation in pericytes. Furthermore, the combination of an optimal dose of lenvatinib and an anti-PD-1 antibody robustly suppressed tumor growth.

**Conclusions:**

Our study proposes a mechanism that explains how the optimal dose of lenvatinib induces vascular normalization and confirms its enhanced synergistic effect with ICBs.

## Introduction

Immune checkpoint blockades (ICBs) immunotherapy has been approved for the treatment of hepatocellular carcinoma (HCC) based on the results of a phase I/II trial (CheckMate 040) (1). Although ICBs have proven effective in some HCC patients with generally acceptable adverse event profiles, their response rate (approximately 20%) has been unsatisfactory (2). Thus, new approaches to improve the clinical effectiveness of ICBs therapy in HCC patients are urgently needed.

Sufficient T-cell infiltration into tumors is a prerequisite for response to ICBs (3, 4), and the interaction with tumor blood vessels is the initial step of T cell accumulation in tumors (5). However, the vasculature in HCC is highly permeable, leaky and tortuous with low perivascular coverage, which impairs blood flow and limits the delivery of oxygen, nutrients, and therapeutics, including immune cells and antibodies (6). Hypoxia and a high level of vascular endothelial growth factor (VEGF) can further induce the production of immunosuppressive molecules by tumor cells and lower the expression of adhesion molecules on vascular endothelium which support immune cell adhesion and transmigration into tumor tissues. In addition, immunosuppressive molecules produced by tumor cells upregulate Fas ligand (FasL) and PD-L1 on vascular endothelium and induce apoptosis of infiltrating T cells (7, 8). Therefore, normalizing the function of the tumor vasculature may be a promising strategy to alleviate hypoxia in the tumor and thereby improve T cell infiltration and immunotherapy.

Inhibition of the VEGFA/VEGFR-2 axis has been developed as the most effective strategy to ‘‘normalize’’ the function of tumor vasculature (9, 10). In fact, it has been suggested that the judicious use of antiangiogenic agents (AAs) can transiently ‘‘normalize’’ the abnormal tumor vasculature (11). Basically, a “normalization window” emphasizes the effect of both the treatment time and dose of AAs on the vascular normalization. Since low doses have little effect on the tumor microenvironment (TME), while high doses can lead to rapid vessel pruning, cause damage to normal tissues and even promote invasion and metastasis (12), the process of normalization needs a balanced dose of AAs to prune away immature, disorganized vessels, and actively strengthen those remaining.

Lenvatinib is the only AA that was found not to be inferior to sorafenib in terms of overall survival as first-line treatment of HCC (12). Its potent inhibition of multiple kinases (VEGF receptors 1–3, FGF receptors 1–4, PDGF receptors α and β, RET, and KIT), contributes to its antitumor effect by suppressing angiogenesis, inhibiting tumor cell proliferation, and inducing apoptosis (13). Recent preclinical studies have also demonstrated its immunomodulatory activity (14, 15). Une et al. (16) reported that lenvatinib induced vascular normalization, improved the TME and increased the radiosensitivity of HCC. A clinical study on unresectable thyroid carcinoma revealed that patients treated with a lower-dose regimen of lenvatinib had a better overall survival (17), suggesting that it may be feasible to optimize the dosage of lenvatinib to improve clinical outcome. However, no studies evaluating the correlation between the dosage of lenvatinib and the degree of tumor vascular normalization in HCC patients have been reported to date.

In this study, we evaluated the effect of increasing doses of lenvatinib on the tumor vasculature and T cells accumulation in different murine models. In addition, we also investigated the mechanism by which different doses of lenvatinib exert different effects on vascular normalization and its synergistic effect when combined with an anti–PD-1 antibody in *in vivo* experiments.

## Materials and methods

### Cell lines

Human umbilical vein endothelial cells (HUVECs) were purchased from iCell Bioscience Inc. (Shanghai, China) and cultured in endothelial cell medium (ScienCell Research Laboratories, Carlsbad, CA, USA) supplemented with 5% fetal bovine serum (FBS), endothelial cell growth supplement (ECGS), and antibiotic (penicillin/streptomycin) solution. HUVECs were passaged no more than 6 times prior to use in experiments. Human brain vascular pericytes (HBVPs) were purchased from Fenghbio Co., Ltd. (Changsha, China) and cultured in Dulbecco’s modified Eagle’s medium (DMEM; Gibco, Thermo Fisher Scientific Inc., Waltham, MA, USA) supplemented with 10% FBS (Gibco, Thermo Fisher Scientific Inc.). PLC/PRF/5 cells were cultured in minimum Eagle’s medium (MEM; Gibco, Thermo Fisher Scientific Inc.) containing 10% FBS and non-essential amino acids (NEAA, Gibco, Thermo Fisher Scientific Inc.). Hep1-6 cells were purchased from the Cell Bank of the Chinese Academy of Sciences (Shanghai, China) and were cultured in DMEM containing 10% FBS.

### Animal models

The effect of lenvatinib on tumor vasculature independent of the host immunity was evaluated in female 4-week-old NOD-Prkdc-(em26cd52) Il2rg(em26Cd22)/Nju (NCG) mice and C57BL/6 mice purchased from the GemPharmatech Co., Ltd. (Nanjing, China). All animal experiments were approved by the Institutional Animal Care and Use Committee of Sun Yat-sen University Cancer Center (SYSUCC; Approval No. L102012021030D). The other materials and methods of animal experiments were shown in supplementary data.

### Immunohistochemistry staining

The immunohistochemistry (IHC) staining procedure used was as described elsewhere1. The following anti-mouse primary antibodies were used: CD31 (Cell Signaling Technology, Danvers, MA, USA), α-SMA (α-Smooth Muscle Actin; Cell Signaling Technology), NG2 (Abcam Plc, Cambridge, UK), VEGFR2 (Cell Signaling Technology). Images of five random fields per sample were acquired on a light microscope (Olympus Corporation, Tokyo, Japan). The positive staining area per field was calculated by Image-pro Plus software (Media Cybernetics, Inc.).

### Immunofluorescence staining

Tissue muti-plex IHC staining was performed using a Pano-Panel IHC Kit (Panovue, Beijing, China) according to the manufacturer’s instructions. Immunofluorescence (IF) staining images were acquired using a Vectra Polaris Imaging System (Akoya Biosciences, Marlborough, MA, USA) and quantitatively analyzed using the HALO software (Indica Labs Inc., Corrales, NM, USA). The following anti-mouse primary antibodies were used: CD31 (Cell Signaling Technology), α-SMA (α-Smooth Muscle Actin, Cell Signaling Technology), NG2 (Abcam Plc), CD8 (Abcam Plc), VCAM1 (Abcam Plc), Ki67 (Abcam Plc), Granzyme B (GranB; Abcam Plc). For immunocytochemistry (ICC) staining, HUVECs were fixed in 4% paraformaldehyde for 20 minutes at room temperature, washed three times with PBS, and then incubated with the corresponding primary antibody at 4°C, overnight. Afterwards, after washing three times with PBS f, the cells were incubated with the appropriate fluorescent dye-conjugated secondary antibody for 1 hour at room temperature, and then nuclear stained with 4’,6-diamidino-2-phenylindole (DAPI) for 10 min. Images were obtained by confocal laser scanning microscopy using a Zeiss LSM880 Confocal Microscope (Carl Zeiss Microscopy GmbH, Jena, Germany). The co-localization analysis of the immunofluorescent markers was performed with the Image J software.

### Flow cytometry analysis

HUVECs were starved in serum-reduced (1%) RPMI 1640 medium overnight and treated with lenvatinib (Selleck Chemicals LLC) at different concentrations for 2 h, followed by 60 ng/ml VEGFA165 (PeproTech Inc., Cranbury, NJ, USA). Then, HUVECs were harvested and washed twice with PBS. Cell-surface staining was performed with APC-conjugated anti-human CD304 (neuropilin-1, NRP1) monoclonal antibody (mAb, 354506; BioLegend Inc., San Diego, CA, USA), phycoerythrin (PE)-conjugated anti-human VEGFR-2 mAb (359904; BioLegend Inc.) at 4°C, for 20 minutes. For intracellular staining, HUVECs were permeabilized with BD Cytofix/Cytoperm kit (BD Biosciences) and stained according to the manufacturer’s instructions. For xenograft-derived immune cell stained, tumor tissues were minced and dissociated using a Tumor Dissociation Kit (Miltenyi Biotec Inc., Auburn, CA, USA) according to the manufacturer’s instructions. The tissue homogenates were then filtered through a 70-µm filter to isolate single cells. Immune cells were identified by CD45, CD3, CD4 and CD8. To examine the expression of Granzyme B by T cells, cells were stimulated with phorbol 12-myristate 13-acetate (PMA) and ionomycin (MilliporeSigma, Burlington, MA, USA) in the presence of the protein transport inhibitor brefeldin A (BioLegend Inc.) for 5 h, and then stained with fluorochrome-conjugated primary anti-Granzyme B antibody. All the antibodies were purchased from Biolegend Inc.

### Western blot and immunoprecipitation analysis

HUVECs for Western blot analysis were washed twice with PBS and then lysed on ice with RIPA buffer (MilliporeSigma) with freshly added protease inhibitor cocktail (Roche AG, Basel, Switzerland). The clarified lysates were separated on 10% sodium dodecyl sulfate/polyacrylamide gel electrophoresis (SDS/PAGE) gels and transferred to polyvinylidene fluoride (PVDF) membranes (MilliporeSigma). The membranes were blocked with 5% nonfat milk and protein bands were reacted with the appropriate primary antibodies by incubating at 4°C overnight. After washing the membranes, the protein bands were reacted with horseradish peroxidase (HRP)-conjugated secondary antibody. Subsequently, the immunoreacted protein bands were visualized using an enhanced chemiluminescence detection system (Bio-Rad Laboratories Inc., Hercules, CA, USA).

Cell lysates for co-immunoprecipitation (co-IP) experiments were prepared by lysing cells in IP Lysis Buffer (Beyotime, Shanghai, China) containing a protease inhibitor cocktail (Beyotime). Then, the clarified lysates were incubated overnight at 4°C with the appropriate primary antibodies according to the specific antibody instruction. Subsequently, the sample-antibody mixtures were rotated together with Protein A/G magnetic beads (MedChemExpress LLC., Monmouth Junction, NJ, USA) for 2 h at 4°C and then washed five times with 0.5% Triton X-100 and collected after magnetic separation. The samples were ultimately eluted 1×SDS-PAGE (Beyotime) sample buffer and further assessed by Western blot analysis.

### HUVECs viability assay

HUVECs viability was determined using the cell counting kit-8 (CCK-8) cell viability assay (Dojindo Laboratories, Kumamoto, Japan). HUVECs (3×103 cells per well) were seeded in flat-bottomed 96-well plates. After incubation for 24 h, the cells were treated with lenvatinib at various concentrations and 60 ng/ml VEGFA165 (PeproTech Inc.) in serum-reduced (1%) RPMI 1640 medium for 72 h. The final concentration of dimethyl sulfoxide (DMSO, as vehicle) was ≤1%. Results were expressed as the mean ± S.E.M. of at least three independent proliferation assays. The dose-response curve and half maximal inhibitory concentration (IC50) values of lenvatinib were calculated busing the GraphPad Prism software (GraphPad Software Inc., San Diego, CA, USA).

### Evans blue permeability assay

HUVECs were seeded on the upper chamber of 0.1% gelatin-coated transwell inserts (0.4 µm). After reaching confluence, HUVECS were treated with lenvatinib at different concentrations for 2 h. Afterwards, albumin-Evans Blue complex (2 mg of Evans Blue/100 mL of EndoGROTM supplemented with 0.5% FBS-0.1% BSA and 60 ng/ml VEGF) was added into the upper chamber and incubated for 90 min. The ratio of Evans Blue in the lower/upper chambers was calculated by measuring the optical density (at 620 nm) of the culture medium harvested from both chambers and used to determine *in vitro* permeability.

### HUVECs-HBVPs three-dimensional co−culture model

HUVECs were transduced with recombinant lentiviruses carrying a red fluorescent protein (RFP) expression plasmid and HBVPs were transduced with recombinant lentiviruses carrying a green fluorescent protein (GFP) expression plasmid. HUVECs-RFP and HBVPs-GFP were co-cultured at a 4:1 ratio in Corning Matrigel Matrix (Corning Inc., Corning, NY, USA), supplemented with extracellular matrix (ECM) medium (ScienCell Research Laboratories) containing lenvatinib (at different concentrations), 20 ng/ml PDGF-BB (PeproTech Inc.) and 60 ng/ml VEGFA165 (PeproTech Inc.). Images were acquired 24 and 48 h after seeding to assess the sprouting capacities of endothelial cells (ECs) by fluorescence microscopy using a 4x objective.

### Cell counting kit-8 cell proliferation assay

HBVPs were seeded in 96-well plates at a density of 1 × 103 cells/well (100 μL/well). After allowing cells to attech for 24 h, HBVPs were treated with lenvatinib at different concentrations, 20 ng/ml PDGF-BB (PeproTech Inc.) and 60 ng/ml VEGFA165 (PeproTech Inc.). At the testing time points (24, 48 and 72 h), 10 μL of CCK-8 solution (Dojindo Laboratories) was added to each experimental well and then incubated for 2 h at 37°C. The absorbance was measured at 450 nm with an automatic microplate reader (Molecular Devices LLC, San Jose, CA, USA).

### Transwell migration assay

HBVPs were starved in serum-reduced (1%) RPMI 1640 medium overnight and treated with lenvatinib at different concentrations, 20 ng/ml PDGF-BB (PeproTech Inc.) and 60 ng/ml VEGFA165 (PeproTech Inc.) for 12 h. After detaching by digestion with trypsin and 0.25% EDTA, HBVPs were resuspended and counted. Subsequently, for the migration assay 6.0 × 104 cells in 200 μL of serum-free medium were placed in the upper compartment of a Transwell chamber (Corning; 24-well insert, pore size: 8 μm). The lower chamber was filled with DMEM containing with 10% FBS as a chemoattractant and then incubated for 12 h. After 12 h, the cells on the upper surface of the membrane were carefully wiped off with a cotton swab. The cells on the lower surface were fixed, stained with 0.1% crystal violet, and washed with PBS. The number of stained cells in five randomly chosen visual fields of each insert were counted under a light microscope. The experiments were performed in triplicate.

### Whole-transcriptome sequencing

Total RNA was isolated with TRIzol and purified using the RNeasy Plus Micro Kit (Qiagen, Hilden, Germany) according to the manufacturer’s protocol. The RNA libraries were then sequenced using the Illumina HiSeq4000 platform (Illumina Inc., San Diego, CA, USA) at the Novogene Genomic Sequencing Center (Beijing, China). The RNA sequencing (RNA-seq) sequence reads were aligned to the mouse reference genome using STAR (v2.5.1b). Differential expression analysis was performed using the DESeq2 R package (1.10.1). The resulting P-values were adjusted using the Benjamini and Hochberg’s method for controlling the false discovery rate. An adjusted p-value (padj) < 0.05 and an absolute fold change of 2 were set as the threshold for significantly differentially expressed genes (DEGs). The alignment results were converted to RNA-seq gene expression measurements as FPKM (fragments per kilobase of exon per million fragments mapped) using HTSeq v0.6.0.

### Statistical analysis

Statistical analyses were performed using SPSS (version 19.0) software (IBM Corporation, Armonk, NY, USA) or GraphPad Prism 6 (GraphPad Software Inc.). Based on the distribution level, data are shown as the mean ± S.D. Independent-sample or paired t test was performed to analyze the difference between the two groups with normally distributed continuous variables. For the comparison among treatment groups in the *in vivo* study, one-way analysis of variance (ANOVA) was performed. In all cases, a two-tailed P-value <0.05 was considered statistically significant, *P <0.05, **P <0.01, ***P <0.001, ****P <0.0001.

## Results

### An optimal dose of lenvatinib has superior effect on tissue perfusion and vascular normalization in an immunodeficient mouse model

**Figure 1:**
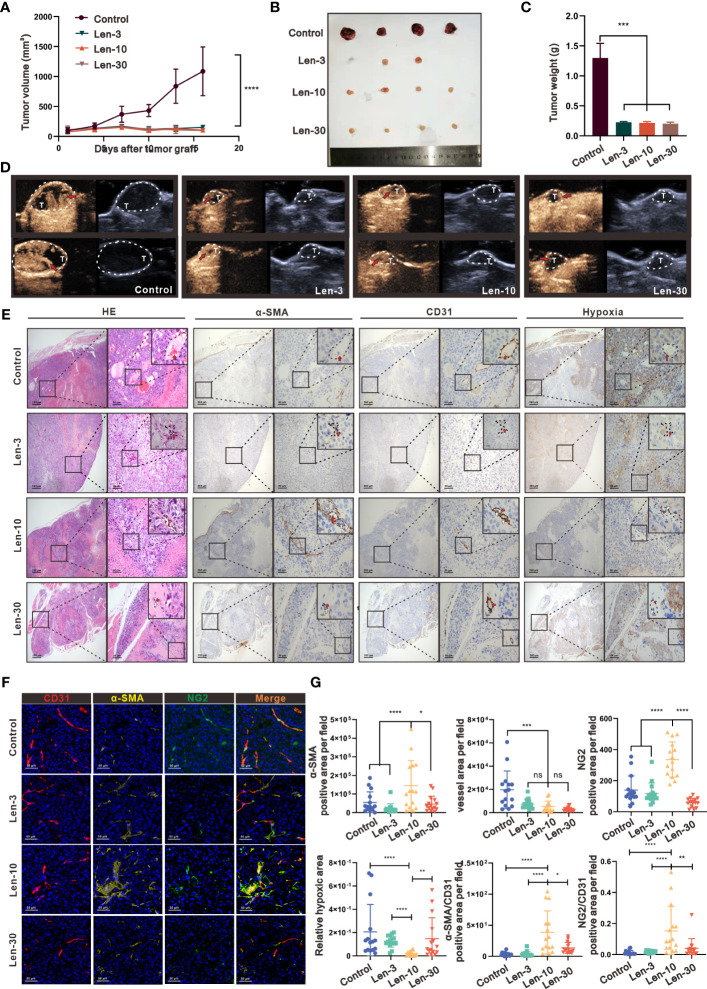
The optimal-dose treatment with lenvatinib had superior effect on tissue perfusion and vascular normalization in an immunodeficient mouse model. **(A)** Volume changes of the subcutaneously implanted tumors in each group beginning at the day of tumor inoculation (n = 5 per group). **(B)** Tumors isolated from mice of each treatment group 16 days after tumor inoculation. **(C)** Tumor weight of mice from each treatment group isolated at day 16 after tumor inoculation. **(D)** Representative perfusion images obtained by CEUS performed on mice from each group after 1 week of treatment. **(E)** Representative images of hematoxylin and eosin (H&E) staining and multi-plex IHC staining of α-SMA, CD31 and pimonidazole in mice from each treatment group. The images are shown at 40×, 200× and 1000× magnification. **(F)** Representative images of the multi-plex IHC staining of α-SMA (yellow), NG2 (green) and CD31 (red) in mice from each group. The images are shown at 200× magnification. **(G)** Statistical graphs of the positive area of α-SMA, CD31, NG2, the relative value of α-SMA/CD31, the relative value of NG2/CD31 and the hypoxia area. α-SMA, α-Smooth Muscle Actin; Len-3, 3 mg/kg/day; Len-10, 10 mg/kg/day; Len-30, 30 mg/kg/day. The error bars represent the mean ± SD. *P < 0.05; **P < 0.01; *** P < 0.001; ****P < 0.0001; ns indicates non-significant.

First, we chose NCG mouse with severe combined immunodeficiency as *in vivo* model to test the efficacy of lenvatinib therapy in modifying the tumor vasculature of HCC to avoid the potential effect of CD4+ T lymphocytes (18) by establishing subcutaneous xenograft tumors derived from the PLC/PRF/5 cell line, which is not sensitive to lenvatinib (19). The administration of lenvatinib was started at 7 days after transplantation of PLC/PRF/5 cells. Previous reports indicated that 3 to 30 mg/kg/day lenvatinib was necessary to achieve anticancer effects in different mouse models (19–21), thus we selected 3, 10 and 30 mg/kg/day as our dose gradients. During the treatment period, the results revealed that compared with the control group treated with CMC, the tumor growth in mice from groups treated with the three different doses was dramatically suppressed with no significant difference among the three groups (**Figures 1A-C**), and a slight decrease in body weight was found only in the Len-30 group (**Figure S1A**). We also assessed the tumor vasculature perfusion and structure after 1 week of treatment, based on the tumor vascular “normalization window” theory used in most studies (10, 22, 23) by real-time contrast-enhanced ultrasonography (CEUS) and IHC staining. According to the perfusion images (**Figure 1D** **)** and videos (Vid. S1), blood perfusion inside the xenograft tumor was dramatically improved only by the dose of 10 mg/kg/day lenvatinib, whereas inside the tumor of the other three groups there were still large non-enhanced areas that lacked blood perfusion. The results suggested that 10 mg/kg/day of lenvatinib had superior effect in inducing the change in the tumor vascular normalization.

As shown by the IHC staining of the tumor vasculature microstructure, after the treatment with lenvatinib, the area of microvessels inside the tumor region dramatically decreased in all the three treated groups (**Figure 1E**). Additionally, the IHC staining results with alpha-smooth muscle actin (α-SMA), which is a classical marker of pericytes, showed that the positive area was significantly higher in the Len-10 group compared with the other three groups. Further evaluation by the multiplex IHC assay with CD31, NG2 and α-SMA directly revealed a typical feature of the mature and stable vasculature with better coverage by pericytes (**Figure 1F**). In addition, the assessment of the degree of hypoxia inside the tumor environment, after injecting a hypoxia detective probe, revealed that the treatments with 10 mg/kg/day lenvatinib significantly alleviated the hypoxia inside the tumor tissue compared with the control group. In addition, we also observed its tendency to alleviate hypoxia in the Len-3 group, but the statistical analysis showed no significant difference between this group and the control group. Noteworthy, there was no significant relief of hypoxia in the group treated with the higher dose of 30 mg/kg/day of lenvatinib (**Figures 1E, G**).

It is well established that VEGFR2 is a highly active receptor that modulates various signaling pathways to promote neovascularization and generate leaky immature blood vessels (24). Therefore, we evaluated the expression of VEGFR2 by IHC, which revealed, as shown in **Figures S1B-D**, markedly reduced expression of VEGFR2 in endothelial cells (ECs) from mice in the Len-10 group. Remarkably, the expression of VEGFR2 in ECs from mice in the Len-30 group was upregulated instead and the relative expression level was even higher than that in the control group. In summary, we confirmed that an escalating dose of lenvatinib exerted a similar inhibitory effect on neovascularization, but a high dose of lenvatinib showed a tendency to aggravate the dysfunction of tumor vasculature.

### An optimal-dose of lenvatinib has superior effects on tissue perfusion and vascular normalization in an immunocompetent mouse model

**Figure 2:**
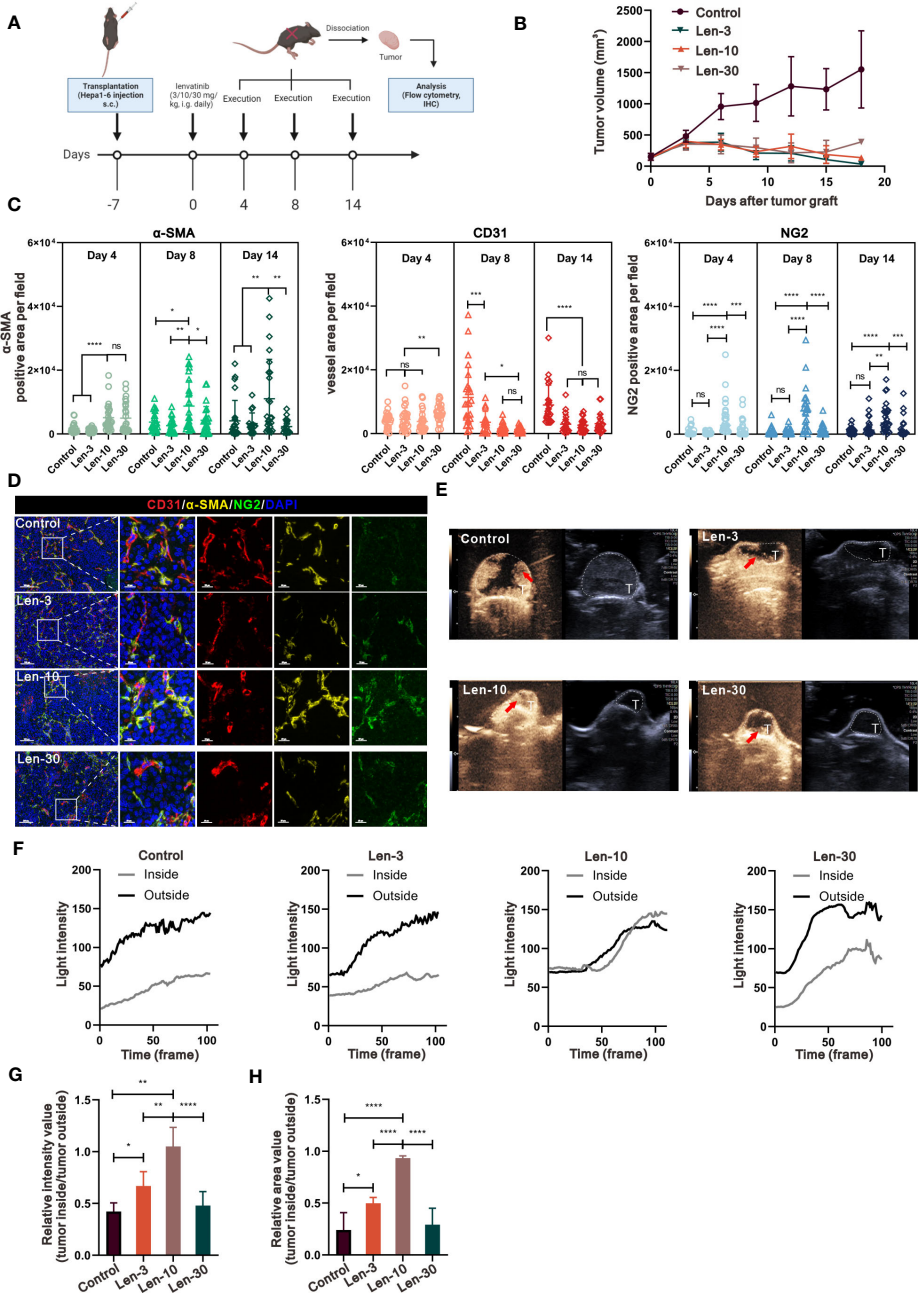
The optimal-dose treatment with lenvatinib had superior effect on tissue perfusion and vascular normalization in immunocompetent mouse model. **(A)** Diagram adapted from “Immunogenicity Assay”, by BioRender.com (2023) and retrieved from https://app.biorender.com/biorender-templates depicting the treatment schedule for the subcutaneous Hepa1-6 tumor model. The xenograft tumors were isolated at day 4, 8 and 14 and were analyzed by IHC and flow cytometry. **(B)** Volume changes of the subcutaneously implanted tumors in each group beginning at the day of tumor inoculation (n = 5 per group). **(C)** Statistical graphs of the positive area of α-SMA, NG2 and CD31 analyzed by IHC on tissue of mice from each group at day 4, 8 and 14. **(D)** Representative multi-plex IHC staining images of α-SMA (yellow), NG2 (green) and CD31 (red) in tissue of mice from of each group. **(E)** Representative perfusion images obtained by CEUS performed on mice from each group at day 8. **(F)** Light intensity inside the tumor region (grey) and outside the tumor region (black) curves examined by CEUS performed on mice from each group. **(G, H)** Statistical graphs of the relative intensity value of tumor inside/outside perfusion **(G)** statistical graphs of the relative area value of tumor inside/outside perfusion **(H)**. The error bars represent the mean ± SD. *P < 0.05; **P < 0.01; *** P < 0.001; ****P < 0.0001; ns indicates non-significant.

We next investigated whether the same effect occurs in the TME of mice with competent immune system in a C57BL/6 mouse model. As shown in **Figure 2A**, we selected the time points at day 4, 8 and 14 to further investigate the effect of variables, such as dose and time, on the dynamic changes in vascular normalization. The analysis of tumor growth inhibition showed that there was no significant difference in tumor volume among the three treatment groups, but their tumor growth was markedly suppressed compared with the control group (**Figure 2B**). Regarding the changes in tumor vasculature, the results showed that the 10 mg/kg/day dose of lenvatinib had superior effect to normalize the tumor vessels at all the time points as indicated by the expression of α-SMA and NG2 (a more specific marker of pericytes) (**Figures 2C**, **S2A-C**). Additionally, their expression levels relative to the expression level of CD31 were dramatically increased in the Len-10 group compared with the other three groups (**Figures S2D, E**). We also found that both the total and relative expression of α-SMA and NG2 reached the highest level on day 8 (**Figures S2D, E**). Moreover, the multiplex IHC analysis of α-SMA, NG2 and CD31 expression, shown in **Figure 2D**, revealed a strong co-localization of α-SMA and NG2 surrounding CD31-stained tumor vessels in the Len-10 group. We also measured blood perfusion inside the tumor by CEUS on day 8 and representative images are shown in **Figure 2E**. The results showed that after the administration of contrast agents only in the Len-10 group the light intensity inside the tumor increased rapidly, following the increase in its intensity outside the tumor. However, the increase of the light intensity inside the tumor in the other three groups was blocked at a significantly lower level than the intensity outside the tumor (**Figure 2F**). In addition, we compared the maximum of the respective intensity value and perfusion area among the four groups, and both parameters showed the same tendency that the Len-10 group had the optimal condition of blood perfusion (**Figures 2G, H**). Overall, we concluded that the dose of 10 mg/kg/day exerted the optimal normalizing effect both in immunodeficient and immunocompetent mouse models.

### The concentration-escalation treatment with lenvatinib reveals different phenotypes of vascular permeability and interaction with pericytes *in vitro*

**Figure 3:**
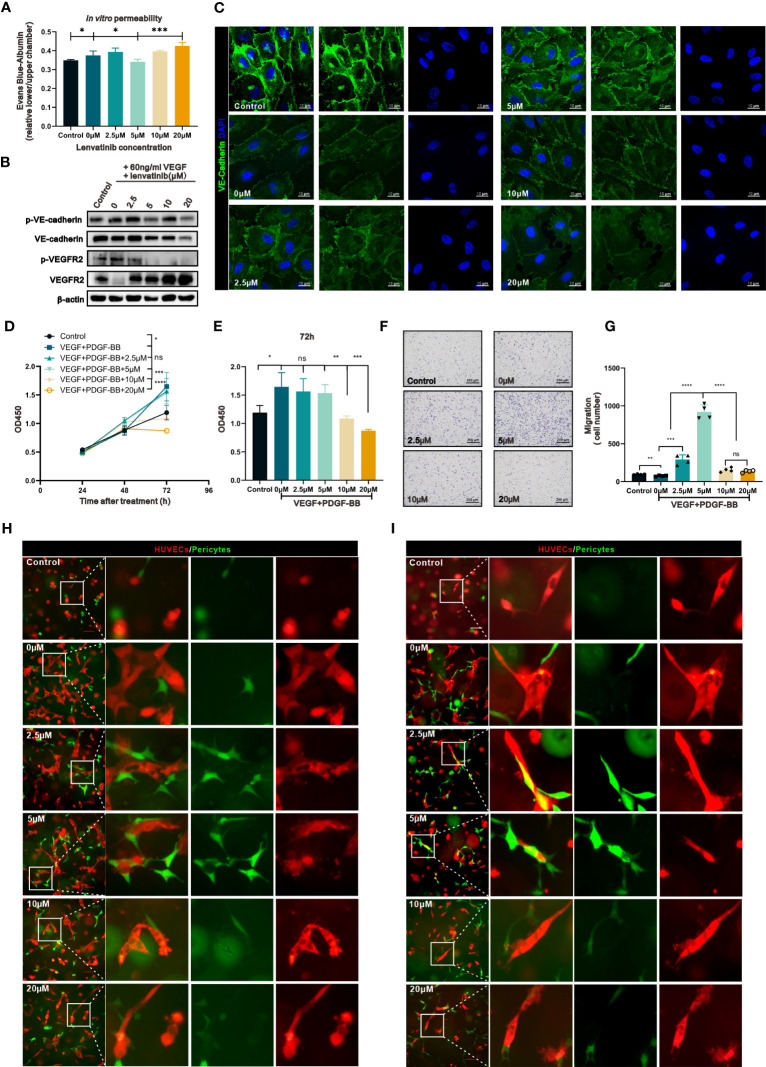
The concentration-escalation treatment with lenvatinib showed different phenotypes of vascular permeability and interaction with pericytes. **(A)** The permeability of the endothelial monolayer was assessed by the passage of Evans blue (EB)/albumin through the upper endothelial monolayer in the lower chamber (n=3 separate experiments). **(B)** Western blot analysis of the phosphorylation of VE-cadherin and VEGFR2 and total expression of VE-cadherin and VEGFR2 in the indicated HUVECs treated with lenvatinib at different concentrations for 2 h, followed by stimulation with 60 ng/ml VEGF for 1 hour. **(C)** Representative IF staining images of VE-cadherin (green) in HUVECs treated with lenvatinib at different concentrations for 2 h, followed by stimulation with 60 ng/ml VEGF for 1 hour. **(D)** CCK-8 assay showing the proliferation of the indicated HBVPs with different treatments at different time points. **(E)** The OD_450_ value of the indicated HBVPs after different treatments for 72 h. **(F)** Representative images of the indicated HBVPs assessed by the Transwell migration assay after different treatments for 72 h. **(G)** Statistical graphs of migrated HBVPs assessed by the Transwell migration assay after different treatments for 72 h. **(H, I)** Fluorescently-labeled pericytes (green) were plated together with red-labeled endothelial cells (ECs) onto Matrigel at a 4:1 (EC: pericyte) ratio, cultured for 24 h **(H)** and 48 h **(I)**, and the resulting vessel-like structures were analyzed by fluorescent microscopy. The error bars represent the mean ± SD. *P < 0.05; **P < 0.01; *** P < 0.001; ****P < 0.0001; ns indicates non-significant.

To investigate the effect of concentration-escalation of lenvatinib *in vitro*, we performed a cell viability assay to evaluate the antiproliferative activity of lenvatinib over a range of escalating concentrations against HUVECs stimulated with 60 ng/ml VEGFA_165_ for 48 h, which simulated the angiogenesis induced by the high level of VEGF produced within the TME. According to the dose-response curve (**Figure S1E**), the half maximal inhibitory concentration (IC_50_) of lenvatinib against HUVECs was 10.72 μM. Therefore, we chose sequential escalating doses of 2.5, 5, 10 and 20 μM of lenvatinib, which were proportional to the IC_50_. We then examined the integrity of vascular junctions using the Evans blue/Albumin permeability assay. The results showed that the treatment with 5 μM lenvatinib successfully blocked the VEGF-induced increased permeation of Evans blue. However, increasing the concentration of lenvatinib to 10 μM and 20 μM failed to further decrease the permeation of Evans blue and aggravated it instead (**Figure 3A**). In order to confirm the impairment of tight junctions resulting in the hyperpermeability of confluent HUVEC monolayer, we used Western blot analysis to measure the expression and phosphorylation of vascular endothelial (VE)-cadherin, which is an essential component of tight junctions between ECs. The results revealed that the phosphorylation of VE-cadherin was upregulated after the stimulation with 60 ng/ml VEGF_165_, indicating the internalization of VE-cadherin and the impairment of tight junctions (25), but its phosphorylation was significantly reduced by 5 μM lenvatinib, while expression of total VE-cadherin remained unchanged. Noteworthy, although the phosphorylation of VE-cadherin was reduced when the concentration of lenvatinib was increased to 10 and 20 μM, the expression of total VE-cadherin was also downregulated (**Figures 3B**, **S3A-B**).

The analysis of the locational distribution of the expressed VE-cadherin by IF staining revealed, as shown by the images in **Figure 3C**, that its expression was localized to the plasma membrane in areas of cell-cell contact, which was disrupted by treatment with VEGFA. After the pre-treatment with lenvatinib at 2.5 and 5 μM, the effect of VEGFA on VE-cadherin was blocked. However, the higher doses of lenvatinib (10 and 20 μM) had the opposite effect, resulting in the disruption of staining continuity of VE-cadherin in areas of cell-cell contact (**Figure S3C**).

Previous research has well established that perivascular cells (PVCs) that surround and support the endothelium, including pericytes and vascular smooth muscle cells, are critical components for the integrity of vessels (26). Thus, we next investigated the effect of different concentrations of lenvatinib on pericytes. We used a cell viability assay to evaluate the effect of lenvatinib (at different concentrations) on the proliferation of HBVPs treated with 60 ng/ml VEGFA_165_ and 20 ng/ml PDGF-BB to simulate the high levels of VEGFA and PDGF-BB secreted into the TME. The results suggested that lenvatinib at 2.5 and 5 μM had no inhibitory effect on the proliferation of HBVPs induced by VEGFA_165_ and PDGF-BB. However, lenvatinib tended to suppress the proliferation of HBVPs at the higher concentrations of 10 and 20 μM (**Figure 3D**). The difference in proliferation among the cells treated with higher and lower concentrations of lenvatinib appeared to be most obvious at 72 h after the stimulation VEGFA_165_ and PDGF-BB (**Figure 3E**). We also evaluated the effects of lenvatinib at different concentrations on the migration of HBVPs stimulated with high levels of VEGFA_165_ and PDGF-BB. After the combined treatment for 12 h, the results revealed that the stimulation with VEGFA_165_ and PDGF-BB slightly promoted the migration ability of HBVPs, which was further increased by the addition of 2.5 and 5 μM lenvatinib, with 5 μM lenvatinib resulting in the optimal migration. However, the number of migratory cells dramatically decreased when the dose of lenvatinib was increased to 10 and 20 μM (**Figures 2F, G**).

In addition, we used a HUVEC/HBVP three-dimensional (3D) co-culture model to analyze their morphology by fluorescent microscopy after culturing for 24 and 48 h. At 24 h of culturing, this co-culture model produced vessel-like and elongated endothelial structures with associated pericytes. In the control co-culture without VEGFA_165_ and PDGF-BB stimulation, no distinct vessel-like networks formed. In the group stimulated with VEGF and PDGF-BB, the vessel-like structures irregularly extended out from the main stalk of the vessels and the pericytes were still loosely associated with ECs both at 24 and 48 h, which resembled the abnormalities of tumor vasculature. In the groups treated with 2.5 and 5 μM lenvatinib, the pericytes were recruited and closely connected with the ECs at 24 h and the appeared to be more closely connected at 48 h. At the same time, the number of vessel-like tubular structures was less and their diameter was smaller, especially at the time point of 48 h. These features resembled the normalized vasculature. In the groups treated with 10 and 20 μM, the vessel-like tubular structures were significantly pruned, along with the loosely associated pericyte (**Figures 2H, I**). Taken together, the above results indicated that lenvatinib at the concentrations of 5 μM had an optimal effect on restoring the integrity of vessels by reinforcing the tight junction between ECs and increasing the coverage by pericytes *in vitro*.

### Lenvatinib showed cross-family compensatory mechanisms and diverse regulatory effects on the interaction of VEGFR2-NRP-1 and NRP-1-PDGFRβ

**Figure 4:**
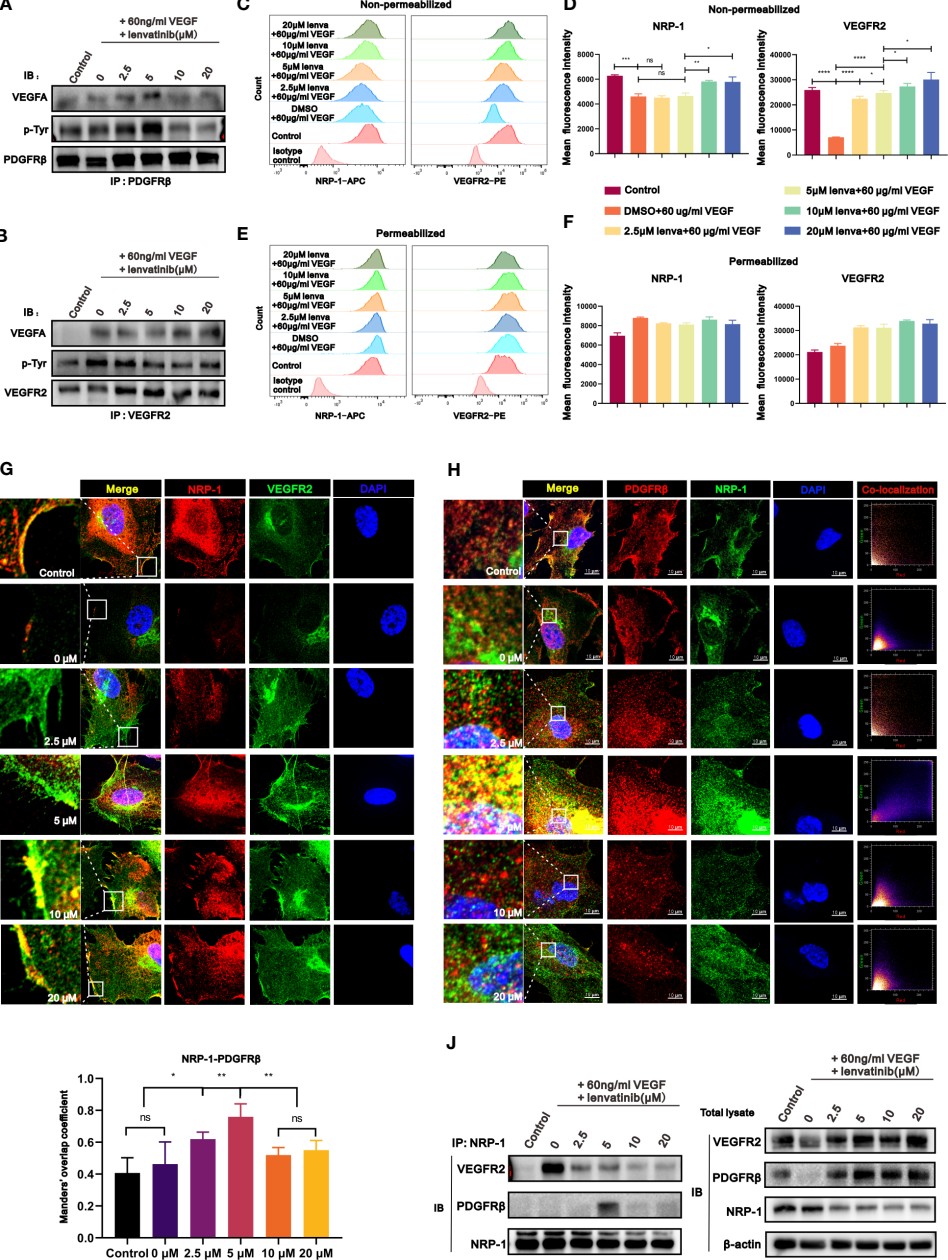
The treatment with lenvatinib showed cross-family compensatory mechanisms and various regulatory effects on the interaction of VEGFR2-neuropilin-1(NRP-1)-PDGFRβ. **(A)** HUVECs were treated with lenvatinib at different concentrations for 2 h, followed by stimulation with 60 ng/ml VEGF for 1 hour and then immunoprecipitated by PDGFRβ. The pulldown samples were analyzed by Western blotting for VEGFA (upper panel), phosphorylated tyrosine (p-Tyr, middle panel), and PDGFRβ (lower panel). **(B)** HUVECs were treated with lenvatinib at different concentrations for 2 h, followed by stimulation with 60 ng/ml VEGF and then immunoprecipitated by VEGFR2. The pulldown samples were analyzed by Western blotting for VEGFA (upper panel), phosphorylated tyrosine (p-Tyr, middle panel), and VEGFR2 (lower panel). **(C-F)** HUVECs were treated with lenvatinib at different concentrations for 2 h, followed by stimulation with 60 ng/ml VEGF. Then the expression of NRP-1 and VEGFR2 in indicated HUVECs was directly analyzed by flow cytometry **(C, D)** or after fixation and permeabilization **(E, F)**. **(G)** Representative images of IF staining of NRP-1 and VEGFR2 in indicated HUVECs treated with lenvatinib at different concentrations for 2 h, followed by stimulation with 60 ng/ml VEGF. **(H)** Representative images and co-localization scatter diagrams of IF staining of NRP-1 and PDGFRβ in indicated HUVECs treated with lenvatinib at different concentrations for 2 h, followed by stimulation with 60 ng/ml VEGF. **(I)** Statistical graph of Manders’ overlap coefficient assessed by co-localization IF staining of NRP-1 and PDGFRβ in indicated HUVECs treated with lenvatinib at different concentrations for 2 h, followed by stimulation with 60 ng/ml VEGF. **(J)** HUVECs were treated with lenvatinib at different concentrations for 2 h, followed by stimulation with 60 ng/ml VEGF, and then subjected to immunoprecipitation with NRP-1 antibody. The total cell lysates were probed with anti-VEGFR2, anti-PDGFRβ, anti-NRP-1 antibodies and anti-β-actin antibodies, while the immunoprecipitates were probed with anti-VEGFR2, anti- PDGFRβ and anti-NRP-1 antibodies. The error bars represent the mean ± SD. *P < 0.05; **P < 0.01; *** P < 0.001; ****P < 0.0001; ns indicates non-significant.

Recently, some studies suggested that a cross-family compensatory mechanism might have a promising effect in regulating angiogenesis, especially through treatment with multi-target small molecule inhibitors (27). Pfister et al. (28) reported that VEGFA can also bind to PDGFRβ and induce its phosphorylation. These findings inspired us to investigate whether the diverse effects of different concentrations of lenvatinib were due to cross-family interaction. We assessed whether the treatment with lenvatinib could regulate the binding of VEGF to PDGFRβ by co-immunoprecipitation (co-IP) analysis. Encouragingly, the treatment with lenvatinib actually promoted the binding of VEGFA to PDGFRβ, and this effect was most evident in the group treated with 5 μM lenvatinib. In addition, the tyrosine-phosphorylation of PDGFRβ was also enhanced (**Figures 4A**, **S3D**), which triggered specific regulatory mechanisms. At the same time, we also observed that the tyrosine-phosphorylation of VEGFR2 was inhibited by lenvatinib in a concentration-dependent manner without affecting the interaction with VEGFA_165_ (**Figures 4B**, **S3E**).

NRP-1 is a multifunctional transmembrane receptor protein that interacts with a variety of activated tyrosine kinase receptors (29). Combined with the above hypothesis of cross-family compensation, these results encouraged us to investigate whether the compensatory binding of VEGF could further influence the interaction of NRP-1 with different tyrosine kinase receptors. We first examined the internalization of NRP-1 and VEGFR2 in HUVECs stimulated with 60 ng/ml VEGF and treated with different concentrations of lenvatinib by flow cytometry, which reflected the activation of their downstream signaling pathways. The results revealed that upon single stimulation of HUVECs with VEGFA, the expression of both the NRP-1 and VEGFR2 on the surface of HUVECs was significantly downregulated. Upon further pre-treatment of HUVECs with 2.5 and 5 μM lenvatinib, the surface expression of NRP1 was still downregulated but the downregulation of surface VEGFR2 was blocked. However, in HUVECs pre-treated with 10 μM and 20 μM lenvatinib, both the downregulation of surface NRP-1 and VEGFR2 were both blocked (**Figures 4C, D**). The measurement of total cell expression of NRP-1 and VEGFR2 after cell permeabilization revealed an upward tendency following the stimulation with VEGF, but there was only a slight difference between the groups treated with lenvatinib (**Figures 4E, F**). Additionally, the subsequent locational distribution analysis of NRP-1 and VEGFR2 by IF staining revealed that NRP-1 and VEGFR2 were located on the membrane of resting HUVECs. After stimulating HUVECs with VEGFA, the fluorescence intensity of immunostained NRP-1 and VEGFR2 on the membrane was immediately diminished. However, pre-treatment with 2.5 and 5 μM lenvatinib led to the recovery of the strong fluorescence signal of immunostained VEGFR2 on the cell membrane, suggesting that the internalization of VEGFR2 was inhibited, while the signal of NRP-1 on the membrane remained diminished. Increasing the concentration of lenvatinib to 10 and 20 μM inhibited the internalization of both NRP-1 and VEGFR2, which persistently remained localized on the membrane after stimulation with VEGFA (**Figures 4G**, **S3F**).

Having found the compensatory binding of VEGF to PDGFRβ, we sought to examine the locational correlation between NRP-1 and PDGFRβ. The results in **Figure 4H** reveal that, after stimulation with VEGFA, both the fluorescence intensity of immunostained NRP-1 and PDGFRβ markedly decreased and there was no significant co-localization between them. Also, although the treatment with lenvatinib recovered their fluorescence intensity, only HUVECs treated with lenvatinib at 5 μM showed strong co-localization of the expression of NRP-1 and PDGFRβ according to the scatter plot results and the analysis of overlap coefficient (**Figures 4H, I**). The co-IP assay further validated their interaction at the protein level, which showed that the stimulation with VEGFA markedly promoted the interaction between VEGFR2 and NRP-1, but their interaction was significantly blocked by the treatment with lenvatinib. Instead, the treatment with 5 μM lenvatinib induced the formation of the NRP-1-PDGFRβ complex, which was consistent with the results of the IF staining (**Figures 4J**, **S3G**). In summary, the treatment with 5 μM lenvatinib concurrently blocked the VEGF-induced interaction between VEGFR2 and NRP-1 and promoted the formation of the NRP1-PDGFRβ complex.

### The NRP1-PDGFRβ complex activated the Crkl-C3G-Rap1 signaling pathway after treatment with lenvatinib

**Figure 5:**
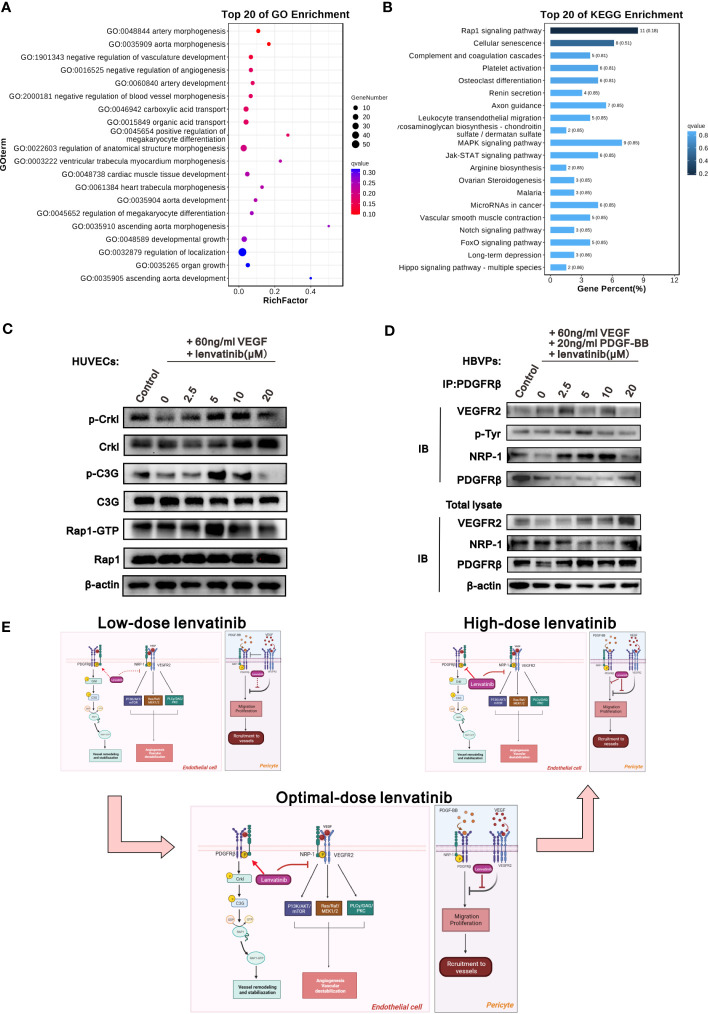
The NRP1-PDGFRβ complex activated Crkl-C3G-Rap1 signaling pathway after treatment with lenvatinib. **(A, B)** HUVECs were treated with 5 μM lenvatinib or DMSO for 2 h, followed by stimulation with 60 ng/ml VEGF, and then subjected to RNA-seq analysis to identify DEGs. The DEGs were analyzed by GO term functional classification **(A)** and KEGG pathway enrichment analysis **(B)**. **(C)** Western blot analysis of the phosphorylation of Crkl, C3G and the activation of Rap1-GTP, as well as the total expression of Crkl, C3G and Rap1 in the indicated HUVECs treated with 5 μM lenvatinib or DMSO for 2 h, followed by stimulation with 60 ng/ml VEGF. **(D)** HBVPs were treated with lenvatinib at different concentrations for 2 h, followed by stimulation with 60 ng/ml VEGF and 20 ng/ml PDGF-BB, and then subjected to immunoprecipitation with PDGFRβ antibody. The total cell lysates were probed with anti-VEGFR2, anti-NRP-1, anti-PDGFRβ and anti-β-actin antibodies, while the immunoprecipitates were probed with anti-VEGFR2, anti-p-Tyr, anti-NRP-1 and anti-PDGFRβ antibodies. **(E)** Schematic diagram depicting the proposed model of the optimal-dose of lenvatinib showing superior effect on the tumor vascular normalization. Low-dose lenvatinib exerts weak inhibitory effect on the neo-angiogenesis mechanism initiated by VEGF binding to VEGFR2 and co-receptor NRP-1, which further activates the PI3K/AKT/mTOR, Ras/Raf/MEK1/2 and PLCγ/DAG/PKC pathways in vascular ECs to promote angiogenesis and vascular destabilization. Likewise, it also has a weak regulatory effect on the NRP1-PDGFRβ-Crkl-C3G-Rap1 axis to mediate vascular remodeling and stabilization. Optimal-dose of lenvatinib exerts strong inhibitory effect on angiogenesis and also induces the formation of the NRP-1-PDGFRβ complex and activates the Crkl-C3G-Rap1 signaling pathway to promote vascular remodeling and stabilization. High-dose lenvatinib has an excessively strong effect on angiogenesis so that it also inhibits the NRP-1-PDGFRβ-Crkl-C3G-Rap1 axis to mediate vascular remodeling and stabilization, resulting in the excessive pruning of functional vessels. In pericytes, low-dose lenvatinib failed to block the binding of VEGFR2 and PDGFRβ, resulting in the inhibition of the tyrosine-phosphorylation of PDGFRβ induced by PDGF-BB to decrease pericyte migration and coverage. An adequate dose of lenvatinib can block the binding of VEGFR2 and PDGFRβ, and further induce the binding of PDGFRβ and NRP-1 to rescue the tyrosine-phosphorylation of PDGFRβ induced by PDGF-BB, which further increases pericyte migration and coverage. High-dose lenvatinib inhibited the proliferation and migration of pericytes.

To investigate the downstream signaling pathway of the NRP1-PDGFRβ complex induced by lenvatinib, RNA-seq analysis was performed on HUVECs treated with different concentrations of lenvatinib and 60 ng/ml of VEGF. We then identified DEGs between HUVECs treated with 5 μM lenvatinib and 0 μM lenvatinib. Gene ontology (GEO) functional classification enrichment analysis of the DEGs identified from the RNA-seq data assigned the DEGs that were significantly enriched in GEO terms to a series of functional groups associated with the regulation of vascular development and morphogenesis (**Figure 5A**), which was consistent with our previous *in vitro* experiments. In addition, the Kyoto Encyclopedia of Genes and Genes pathway enrichment analysis showed that the top enriched pathway was the Rap1 signaling pathway (**Figure 5B**).

We also examined the activation of the Crkl-C3G-Rap1 axis, which is a typical signaling pathway that regulates the activity of Rap1 (30, 31). The western blot analysis results in **Figure 5C** reveal that the activation of Crkl, C3G and Rap1 showed the same tendency for all six treatment groups, and the activation of all three proteins was highest in the group treated with 5 μM lenvatinib (**Figures 5C**, **S4A-C**). In addition, we treated the HBVPs with lenvatinib at different concentrations followed by stimulation with 60 ng/ml VEGFA_165_ and 20 ng/ml PDGF-BB. The IP assay showed that only the treatment of 5 μM lenvatinib was able to significantly block the interaction between VEGFR2 and PDGFRβ, but it promoted the interaction between NRP-1 and PDGFRβ. Additionally, the tyrosine-phosphorylation of PDGFRβ was clearly increased by lenvatinib (**Figures 5D**, **S4D**). Based on these results, we propose a schematic model to illustrate the mechanism of the various effects of the treatment with lenvatinib at different doses on vascular normalization (**Figure 5E**).

### The optimal-dose treatment with lenvatinib had superior effect on immune cell infiltration

**Figure 6:**
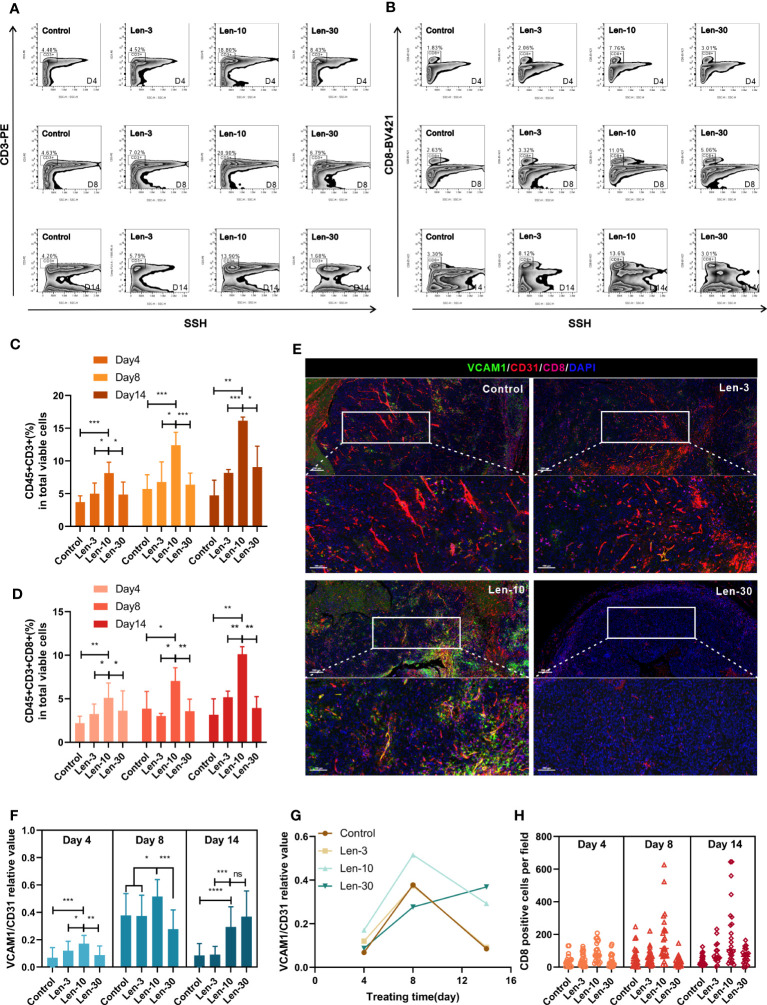
The dose-escalation treatments of lenvatinib showed a lack of dose-dependent effect by the escalating dose of lenvatinib on immune infiltration **(A, B)** Representative flow cytometry analysis of the CD3+ T cells **(A)** and CD8+ T cells **(B)** in mice from each group on day 4, 8 and 14 after the treatment. **(C, D)** Statistical graphs of CD45+CD3+ percent **(C)** and CD45+CD3+CD8+ percent **(D)** in total viable cells assessed by flow cytometry in mice from each group on day 4, 8 and 14 after the treatment. **(E)** Representative multi-plex IHC staining images of VCAM1 (green), CD31 (red) and CD8 (pink) in mice from each group on day 8 after the treatment. **(F)** Statistical graphs of relative values of VCAM1/CD31 positive area assessed by multi-plex IHC staining in mice from each group on day 4, 8 and 14 after the treatment. **(G)** The change trendline of VCAM1/CD31 relative values in mice from each group during the treatment. **(H)** Statistical graphs of the number of CD8+ T cells in mice from each group on day 4, 8 and 14 after the treatment. The error bars represent the mean ± SD. *P < 0.05; **P < 0.01; *** P < 0.001; ****P < 0.0001; ns indicates non-significant.

We next evaluated the effect of the optimal dose of lenvatinib on the infiltration of different immune cells inside the tumor. Flow cytometry analysis revealed that the number of infiltrated leukocytes inside the subcutaneous tumors significantly increased in the Len-10 group compared with the other three groups at all checked time points (day 4, 8 and 14) (**Figures S3A-D**). Regarding functional lymphocytes, both the proportion of CD45+CD3+ cells and CD45+CD3+CD8+ cells was significantly increased in the Len-10 group compared to the control, Len-3 and Len-30 groups at all the checked time points, and the difference was more evident at day 8 and 14 (**Figures 6A-D**). Meanwhile, the proportion of intratumoral CD45+CD4+ showed no difference among these four groups at day 4. Additionally, at day 8 of treatment, the Len-10 group had the largest proportion of infiltrated CD45+CD4+ cells as CD3+ and CD8+ T cells. However, the number of infiltrated CD45+CD4+ cells significantly increased in the Len-30 group, but their number in the other three groups decreased (**Figures S5E-H**).

It has previously been established that the infiltration of immune cells is closely related to the expression density of vascular cell adhesion molecule 1 (VCAM1) in vascular ECs (32). We evaluated the expression levels of VCAM1, CD31 and CD8. At days 4 and 8 of the treatments, the area ratio of VCAM1 *versus* CD31 significantly increased in the Len-10 group compared to the other three groups (**Figure 6E**). Noteworthy, the area ratios of the control, Len-3 and Len-10 groups collectively decreased while the area ratio of the Len-30 group markedly increased and surpassed the ratio of the Len-10 group at day 14 (**Figures 6F, G**, **S6A**). However, the number of infiltrated CD8+ T cells was always the highest in the Len-10 groups at all checked time points (**Figure 6H**), which suggested that the upregulated expression of VCAM1 early in the treatment benefited immune cell infiltration. These results showed that, in contrast to the higher dose of 30 mg/kg/day, the 10 mg/kg/day dose of lenvatinib had an optimal effect on inducing vascular normalization and increasing the immune infiltration *in vivo*.

### Combined therapy with the optimal dose of lenvatinib and anti-PD-1 markedly suppressed tumor growth

**Figure 7:**
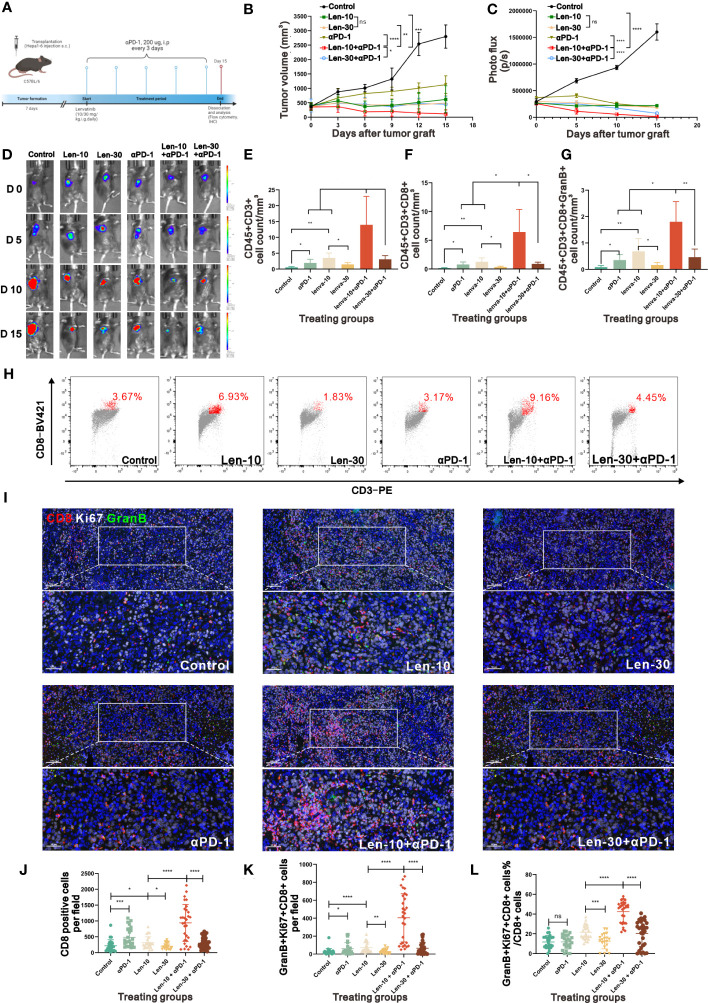
The combined therapy of PD-1 antibody and 10 mg/kg/day lenvatinib strongly suppressed tumor growth. **(A)** Diagram adapted from “Mouse Experimental Timeline”, by BioRender.com (2023) and retrieved from https://app.biorender.com/biorender-templates depicting the treatment schedule for the combined therapy of PD-1 Antibody and lenvatinib in the subcutaneous Hepa1-6 tumor model. **(B)** Volume changes in the subcutaneously implanted tumors in mice from each group beginning at the day of tumor inoculation (n = 5 per group). **(C, D)** Photo flux statistics and representative images reflecting the growth of tumors measured using an *in vivo* imaging system at 0, 5, 10 and 15 days after the treatment; n = 5 for each group. **(E-G)** Statistical graphs of CD45+CD3+ T cell count **(E)**, CD45+CD3+CD8+ T cell count **(F)** and CD45+CD3+CD8+GranB+ T cell count **(G)** standardized by tumor volume measured by flow cytometry in mice from each group on day 15 after the treatment. **(H)** Representative flow cytometry analysis of the CD3+CD8+ T cells in mice from each group on day 15 after the treatment. **(I)** Representative multi-plex IHC staining images of GranB (green), Ki67 (white) and CD8 (red) in mice from each group on day 15 after the treatment. **(J-L)** Statistical graphs of CD8+ T cells number, GranB+Ki67+CD8+ T cell number and the percent of GranBGzmb+Ki67+ T cell number in total CD8+ T cell number in mice from each group on day15 after the treatment. Len-10, 10 mg/kg/day; Len-30, 30 mg/kg/day; αPD-1, anti-PD-1 antibody; Len-10+αPD-1, 10 mg/kg/day+anti-PD-1 antibody; Len-30+αPD-1, 30 mg/kg/day+anti-PD-1 antibody. The error bars represent the mean ± SD. *P < 0.05; **P < 0.01; *** P < 0.001; ****P < 0.0001; ns indicates non-significant.

We also evaluated the PD-1 expression in the tumor samples from the control, Len-10 and Len-30 groups at different time points by multiplex IHC staining of PD-1 and CD8. The results revealed that both the number of PD-1+ cells and PD-1+CD8+ cells was higher in the Len-10 group than in the Len-30 group at all checked time points (**Figures S7B-E**). These findings led us to hypothesize that the combination treatment with 10 mg/kg/day lenvatinib and anti-PD-1 antibody could produce enhanced antitumor effect in the murine model. To test this hypothesis, we treated the tumor-bearing mice with two doses (10 or 30 mg/kg) of lenvatinib or CMC treatment once a day, combined with the administration of anti-PD-1 antibody (100 μg, i.p.) or IgG control once every three days (**Figure 7A**) to investigate their synergetic effect.

As hypothesized, the combined treatment with lenvatinib and anti-PD-1 antibody suppressed tumor growth compared with either the monotherapy group or control group. In addition, tumor growth was more prominently inhibited and even regressed by the 10 mg/kg/day lenvatinib plus anti-PD-1 antibody treatment compared to the higher dose (30 mg/kg/day) combination treatment (**Figures 7B**, **S7B**). The photo flux statistics and representative fluorescence images of live imaging also showed the same tendency (**Figures 7C, D**). No statistical difference in body weight was found among all groups (**Figure S7A**), suggesting that the combination treatment had no noticeable toxicity.

Moreover, the examination of the number and functions of infiltrated lymphocytes in the harvested tumor samples at the end of the therapy revealed that the Len-10+αPD-1 group had a better immune-supportive environment, accompanied by more CD45+CD3+ T cells, CD45+CD3+CD8+ T cells and CD45+CD3+CD8+GranB+ activated T cells (**Figures 7E-G**). Representative flow cytometry graphs showed clearly different clusters of CD3+CD8+ inside the tumors from the six groups (**Figure 7H**). In addition, multiplex IHC staining of CD8, Ki67 and GranB showed that both the number of CD8+ cells and CD8+Ki67+GranB+ cells per field were highest in the Len-10+αPD-1 group (**Figures 7I-K**). At the same time, we assessed the stability of tumor vessels by IHC. The results showed that there were more pericytes surrounding the vessels in the tumor tissue from the tumor-bearing mice treated with 10 mg/kg/day lenvatinib as well as 10 mg/kg/day Lenvatinib plus anti-PD-1 antibody, while the treatment of anti-PD-1 antibody demonstrated no effect on the vascular normalization in our study (**Figures S8A-D**)

**Figure 8:**
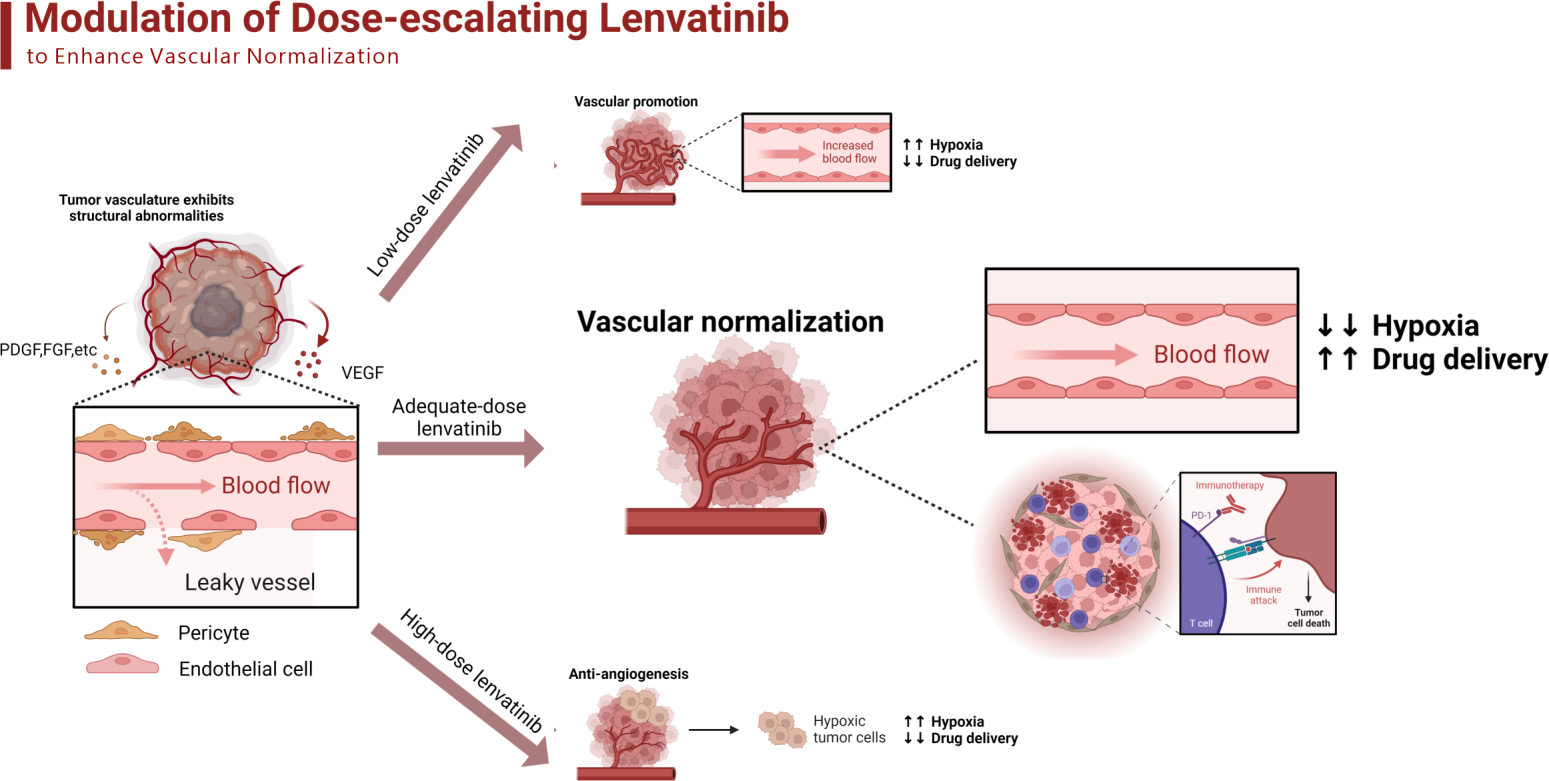
The schematic diagram adapted from “Modulation of Tumor Blood Vessels”, by BioRender.com (2023) and retrieved from https://app.biorender.com/biorender-templates of dose-escalating lenvatinib to enhance vascular normalization and elevated the efficacy of ICBs. The tumor cells secret different kinds of growth factors such as VEGF, PDGF and FGF to promote angiogenesis, which results in the structural abnormalities of tumor vasculature. The low-dose lenvatinib fails to effectively inhibit the abnormal angiogenesis so there are still numerous dysfunctional vessels to limit the transportation of oxygen and drugs. The adequate-dose lenvatinib effectively prune the abnormal vessels and promoted the normalization of the remaining, which improves the immunosuppressive TME and elevated the efficacy of ICBs. The high-dose lenvatinib excessively prunes the functional vessels inside the tumor and results in the shortage of blood supply, which aggravates the hypoxia and perfusion.

Additionally, the analysis of the proportion of Ki67+GranB+ cells in total CD8+ cells revealed that the combination treatment with anti-PD-1 antibody and lenvatinib upregulated the proportion of Ki67+GranB+ cells in CD8+ T cells, with the best effect being obtained with the 10 mg/kg/day dose of lenvatinib. However, the monotherapy with anti-PD-1 antibody failed to increase the proportion of Ki67+GranB+ cells compared with the control group (**Figure 7L**). A summary of the treatment with the dose-escalating lenvatinib to enhance vascular normalization and increase the efficacy of ICB immunotherapy is shown in **Figure 8**.

## Discussion

Many factors have been reported to limit the curative effect of ICBs, including the TME, host genomics, systemic immunity, microbiota, *etc.* Among them, the TME is the factor that has generated the most research and debate (33). This study focused on the tumor vasculature, and examined whether a strategy that effectively optimizes the dose of lenvatinib to further promote the normalization of tumor vasculature could potentially be used to improve the effectiveness of ICBs.

To date, some research has confirmed the definitive effect of RTK inhibitors on induction of vascular normalization and enhancement of the efficacy of immunotherapy. For example, Zhao et al. reported that treatment with apatinib normalized the vasculature, increased infiltration of CD8+ T cells in lung cancer tumors, and the combination of apatinib with anti-PD-L1 antibody significantly suppressed tumor growth (34). Additionally, axitinib was also shown to lead to increased proximity between PVCs and ECs both in murine Lewis lung carcinoma (LLC) (35) and in a pancreatic insulinoma model (36). Other inhibitors, such as sunitinib and cediranib, have also been found to cause similar change in tumor vasculature in glioma xenograft mouse models (37, 38). However, there is no concrete research on the optimal dose of lenvatinib to improve vascular normalization and avoid the recurrence of resistance. In this study, we confirmed that an escalating dose of lenvatinib exerted a similar inhibitory effect on neovascularization, but a high-dose of lenvatinib (30 mg/kg/day) showed a tendency to aggravate the tumor vasculature dysfunction. Only the optimal dose (10 mg/kg/day) significantly increased the coverage with pericytes, alleviated hypoxic TME and increased vascular perfusion. Moreover, similar results were also obtained in a C57BL/6 mouse model with an immunocompetent condition. These findings suggested that only an adequate dose of lenvatinib could achieve optimal efficacy to change the abnormal vasculature rather than recklessly prune the vessels. The *in vitro* experiments revealed that the optimal-concentration (5 μM) of lenvatinib had a superior effect on restoring the integrity of vessels by strengthening tight junctions between HUVECs and reducing the VEGF-induced leakiness. Moreover, the optimal-concentration lenvatinib also promoted the migration of HBVPs to the vessel periphery and increased the coverage of HBVPs around the vasculature. These findings are in line with the *in vivo* findings about vascular normalization.

It is well established that TKIs directly inhibit the tyrosine kinase domain of receptors but do not interfere with the binding of ligands to their receptors. In addition, the multi-target characteristic is manifested in the broad spectrum of activity inhibiting several members of the kinases simultaneously with differing potencies (26). VEGFR2 was the most sensitive receptor with the lowest IC_50_, while PDGFRs and EGFRs were less sensitive among the main target receptors of lenvatinib (39). Remarkably, our study showed that the most obvious interaction between VEGF and PDGFRβ occurred during the treatment with 5 μM lenvatinib followed by 60 ng/ml VEGF, which suggested that the adequate concentration of lenvatinib initiated the cross-family compensatory mechanism to induce the phosphorylation of PDGFRβ (28, 40).

Previous studies found that NRP-1 plays an unessential role in the proliferation of ECs mediated by VEGF and indicated that NRP-1 is a regulator of angiogenesis instead of an inducer of vasculogenesis (29, 41). Moreover, Pan et al. (42) found that blocking NRP1 function inhibited vascular remodeling and decreased the coverage of tumor vessels by pericytes. Our study also indicated that the treatment with lower concentration of lenvatinib (2.5 and 5 μM) could rapidly block the internalization of VEGFR2, but failed to block the internalization of NRP1, suggesting that NRP-1 exerted its regulatory effect independently of VEGFR2 as internalization is a typical way by which tyrosine kinase receptors transmit activation signals to the cytoplasm. We further confirmed the concurrent formation of the NRP-1-PDGFRβ complex. Based on previous study indicating that VEGF binds to PDGFRβ as a compensatory mechanism and NRP-1 acts as a co-receptor of PDGFRβ to regulate cell morphology (43), we concluded that the treatment with 5 μM of lenvatinib concurrently blocked the VEGF-induced interaction between VEGFR2 and NRP1 and promoted the formation of the NRP1-PDGFRβ complex.

It has been reported that tyrosine-phosphorylated PDGFRβ could bind to the SH2 domain of Crk family adaptors, which is required for the regulation of cellular adhesion, migration and proliferation (44). In addition, some studies have verified that the activation of Crk adaptors could lead to further phosphorylation of C3G (CrkSH3-domain-binding guanine-nucleotide releasing factor, where SH3 stands for Src homology region 3 domain), which is a typical guanine nucleotide-exchange factors (GEFs) to switch on the Rap1 signaling pathway by regulating the GDP-GTP cycle (30, 31). Our results of Western blot analysis in HUVECs treated with 5 μM lenvatinib and 60 ng/ml VEGF found upregulated phosphorylation of the Crkl adaptor, C3G and activation of Rap1 (45). Therefore, we surmise that only the appropriate concentration of lenvatinib can activate the specific signaling pathway to regulate the vascular remodeling process and transform the abnormal vasculature through a cross-family compensatory mechanism among different tyrosine kinase receptors, ultimately leading to the activation of the Crkl-C3G-Rap1 signaling pathway.

Vascular normalization could increase the infiltration of cytotoxic T cells into tumors by upregulating the expression of the leukocyte-adhesion molecules VCAM1 and E-selectin in tumor endothelium (26). In this study, we observed significantly increased infiltration of CD8+ cytotoxic T cells in the Len-10 group, and the immune infiltration increased over time, which is consistent with the vascular normalization tendency observed *in vivo*. Additionally, we also observed that the expression of VCAM1 was significantly higher in tumor endothelium of the Len-10 group in the early treatment stages. However, it is worth noting that the expression of VCAM-1 decreased in the control, Len-3 and Len-10 groups in the late treatment stage (day 14) except in the Len-30 group. In contrast, the expression of VCAM1 dramatically increased and reached its highest level among all four treatment groups. This phenomenon can be explained by the finding that the sustained over expression of VCAM1 is a hallmark of senescent ECs related to the loosening of EC junctions, which facilitates tumor cell transmigration through the endothelial barrier and ultimately accelerates metastasis (46). Therefore, the indiscriminate dosing of lenvatinib might result in adverse long-term effects. Our study determined the optimal therapeutic efficacy to suppress tumor growth of the lower-dose combination therapy of 10 mg/kg/day lenvatinib and anti-PD-1 antibody compared with the higher-dose (30 mg/kg/day) combination therapy, which further highlighted the importance of using the proper dose of lenvatinib when used in combination with ICB immunotherapy.

In summary, our study indicated that lenvatinib, a multi-target TKI inhibitor when administrated at an adequate dose, could further optimize the immature and dysfunctional tumor vasculature as well as increase the immune cell infiltration to remodel the TME. The change in tumor vasculature was mainly achieved by inducing the VEGF compensatory binding mechanism to form the NRP-1-PDGFRβ complex and activate the Crkl-C3G-Rap1 signaling pathway in HUVECs. Additionally, the optimized tumor vasculature and TME improved the sensitivity to ICBs therapy. Our study emphasizes the need to carefully design the dosage of lenvatinib to achieve the optimal therapeutic efficacy, especially when the combination with ICBs is considered, since the benefits of lenvatinib are not dose dependent. In the future, additional evaluation in more mouse experimental systems and randomized trials in human is warranted.

## Data availability statement

The original contributions presented in the study are included in the article/**Supplementary Materials**, further inquiries can be directed to the corresponding author/s.

## Ethics statement

The animal study was reviewed and approved by Institutional Animal Care and Use Committee of Sun Yat-sen University Cancer Center.

## Author contributions

Conception and design: JX, TX, JY, ZG, MS. Development of methodology: JY, ZG, MS, YH, DO. Acquisition of data: JY, ZG, MS, QW, YLH, YL. Analysis and interpretation of data: JY, ZG, MS, QP, JZ, CY, MD. Writing, review, and/or revision of the manuscript: JY, MS, JX, TX. Administrative, technical, or material support: ZG, JH, QP, YT, HC, DW.
