# Supplementary material for: Lenvatinib improves anti-PD-1 therapeutic efficacy by promoting vascular normalization via the NRP-1-PDGFRβ complex in hepatocellular carcinoma
Source: Front Immunol. 2023 Jul 21;14:1212577. doi: 10.3389/fimmu.2023.1212577 (PMC10400764; doi:10.3389/fimmu.2023.1212577)
Supplement: Supplementary file 1 [file DataSheet_1.zip › Supplementary data.pdf]

Supplementary Figures

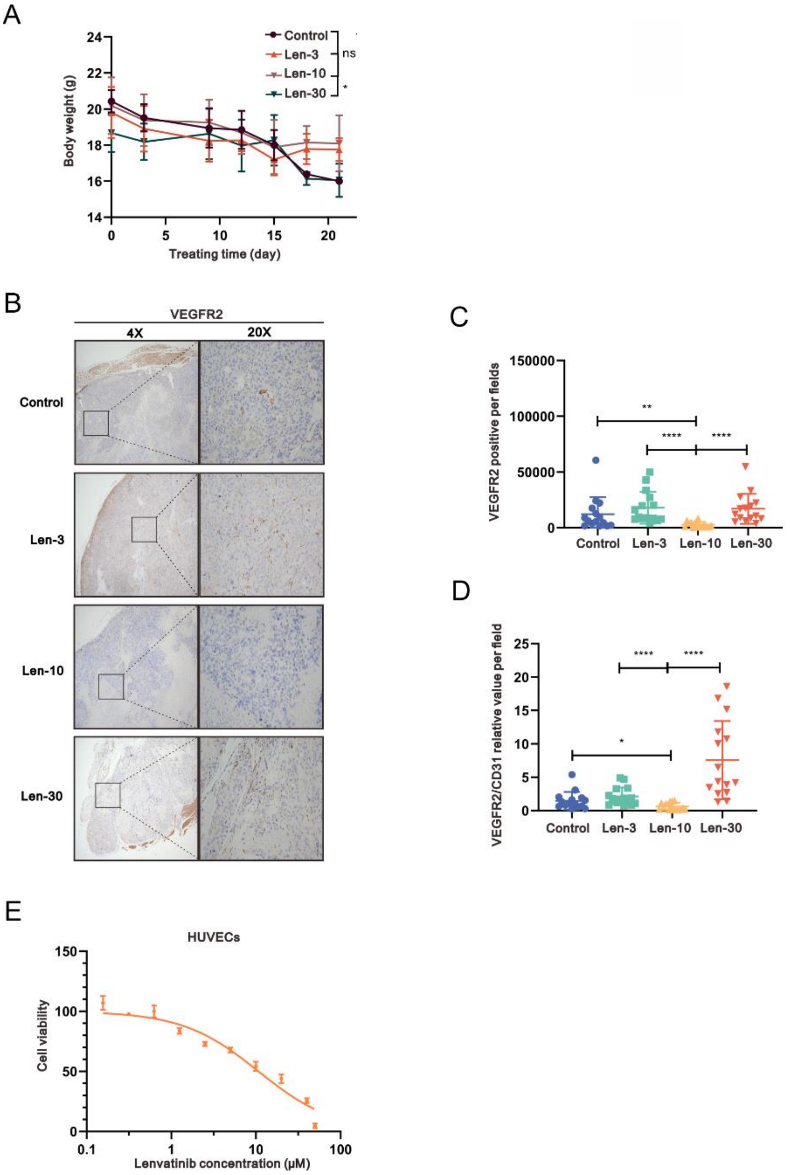

Fig. S1 (**A**) Body weight changes of subcutaneously implanted tumors in mice from each group beginning at the day of tumor inoculation (n = 5 per group). (**B**) Representative IHC staining images of VEGFR2 in mice from each group. (**C-D**) Statistical graphs of VEGFR2 positive area (**C**) and VEGFR2/CD31 relative area value S (**D**) in mice from each group assessed by IHC staining. (**E**) Concentration-dependent curve of HUVECs treated with different concentrations of lenvatinib.

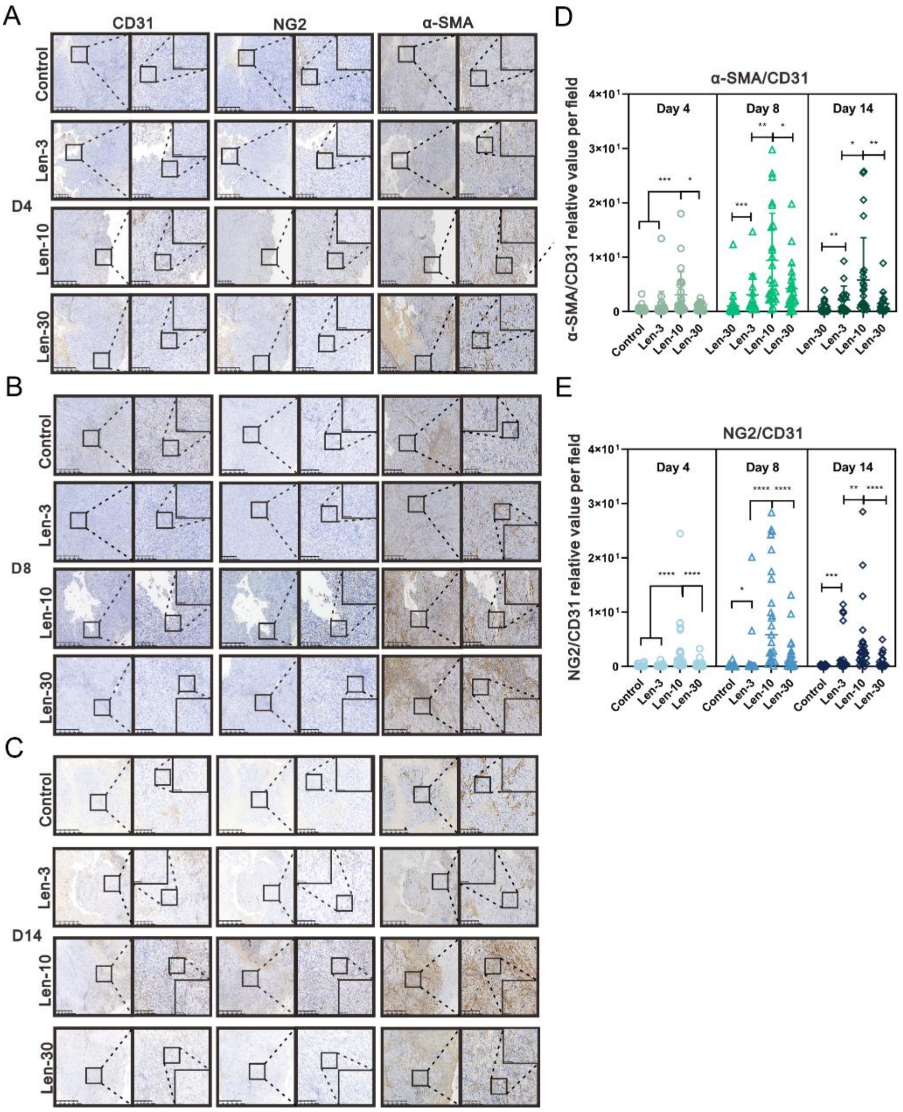

Fig. S2 (**A-C**) Representative IHC staining images of CD31, NG2 and α-SMA in mice from each group on day 4 (**A**), day 8 (**B**) and day 14 (**C**) after the treatment. (**D-E**) Statistical graphs of the relative area value of α-SMA/CD31 (**D**) and NG2/CD31 (**E**) in mice from each group at day 4, 8 and 14.

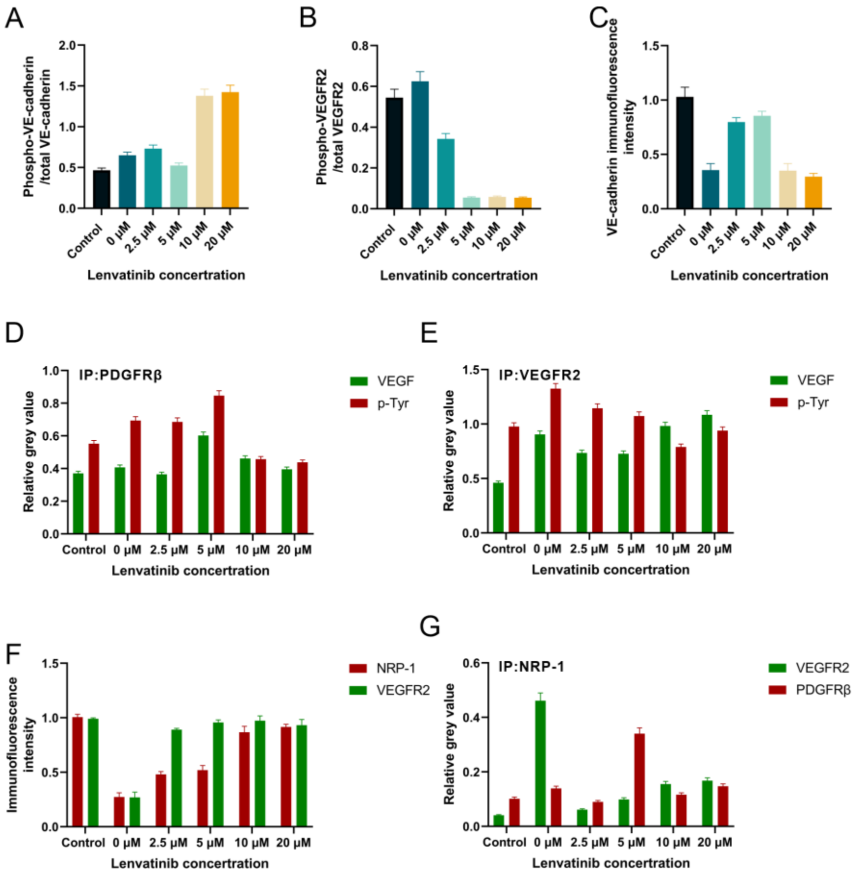

Fig. S3 (**A-B**) Quantification graphs of the expression of phosphorylated VE-cadherin (**A**) and VEGFR2 (**B**) in HUVEC cells treated with lenvatinib at different concentration measured. (**C**) Quantification graphs of IF staining of VE-cadherin (green) in HUVECs treated with lenvatinib at different concentrations for 2 h, followed by stimulation with 60 ng/ml VEGF for 1 hour. (**D-E**) HUVECs were treated with lenvatinib at different concentrations for 2 h, followed by stimulation with 60 ng/ml VEGF for 1 hour and then immunoprecipitated by PDGFRβ (**D**) and VEGFR2 (**E**) respectively. The quantification graphs of pulldown samples were analyzed by western blotting for VEGFA, phosphorylated tyrosine (p-Tyr), PDGFRβ and VEGFR2. (**F**) Quantification graphs of IF staining of NRP-1 and VEGFR2 in indicated HUVECs treated with lenvatinib at different concentrations for 2 h, followed by stimulation with 60 ng/ml VEGF. (**G**) HUVECs were treated with lenvatinib at different concentrations for 2 h, followed by stimulation with 60 ng/ml VEGF, and then subjected to immunoprecipitation with NRP-1 antibody. The quantification graphs of pulldown samples were analyzed by western blotting for PDGFRβ and VEGFR2.

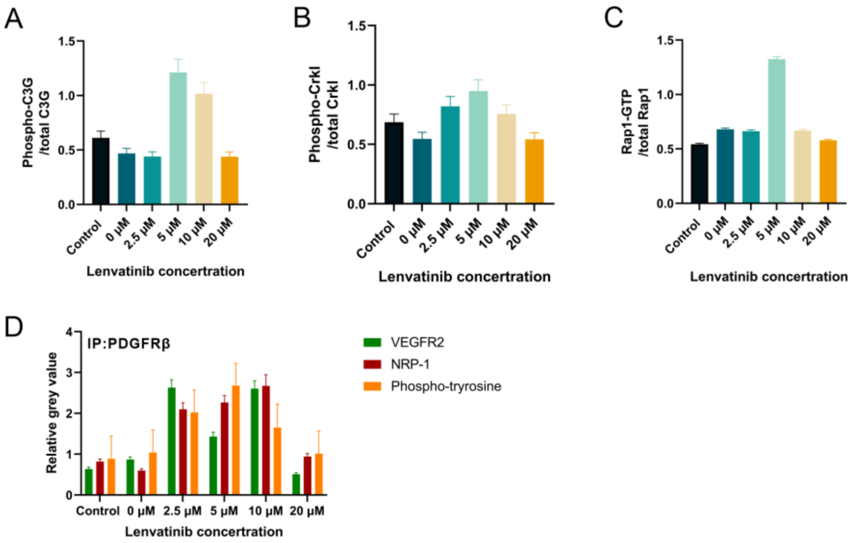

Fig. S4 (**A-C**) Quantification graphs of the expression of phosphorylated C3G (**A**), Crkl (**B**) and Rap1-GTP (**C**) in HUVEC cells treated with lenvatinib at different concentration measured. (**D**) HBVPs were treated with lenvatinib at different concentrations for 2 h, followed by stimulation with 60 ng/ml VEGF and 20 ng/ml PDGF-BB, and then subjected to immunoprecipitation with PDGFRβ antibody. The quantification graphs of pulldown samples were analyzed by western blotting for VEGFR2, NRP-1 and p-Tyr.

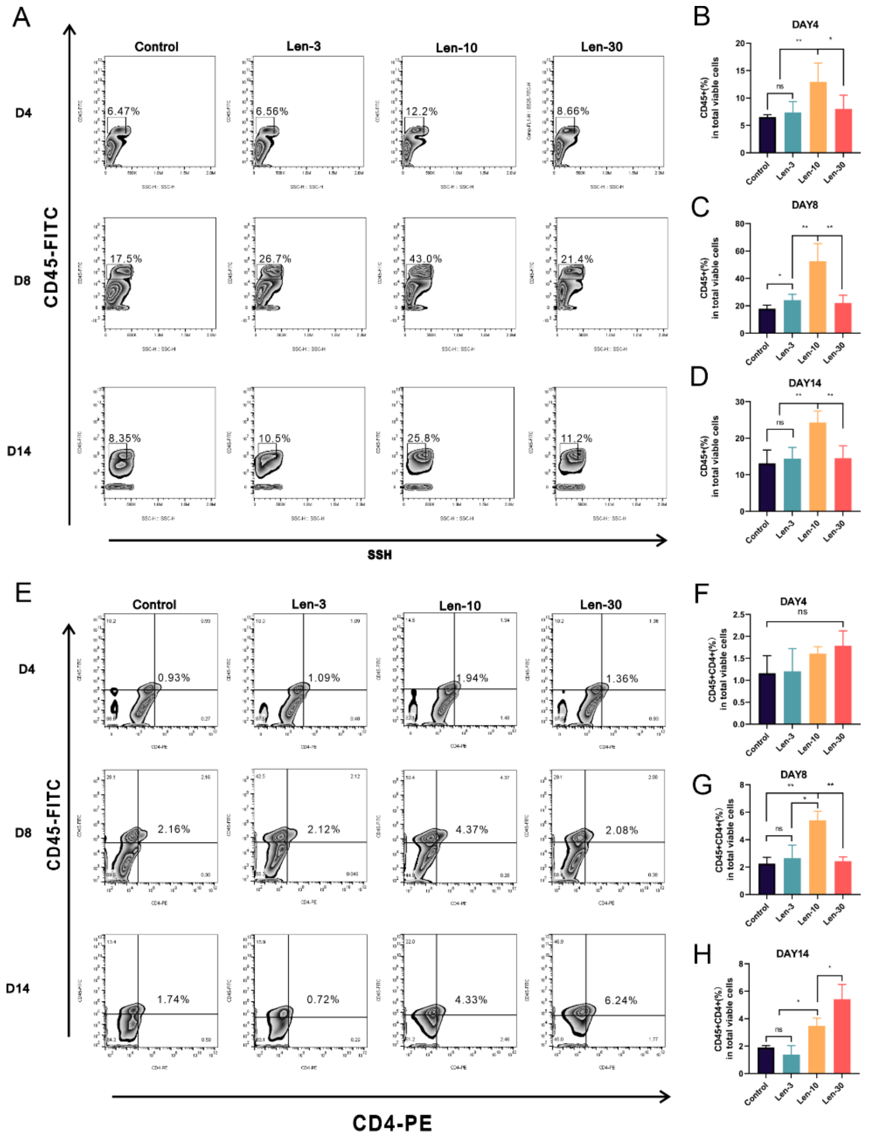

Fig. S5 (**A**) Representative flow cytometry analysis of the CD45+ cells in mice from each group on day 4, 8 and 14 after the treatment. (**B-D**) Statistical graphs of CD45+ cells percent in total viable cells assessed by flow cytometry in mice from each group on day 4 (**B**), day 8 (**C**) and day14 (**D**) after the treatment. (**E**) Representative flow cytometry analysis of the CD45+CD4+ T cells in mice from each group on day 4, 8 and 14 after the treatment. (**F-H**) Statistical graphs of CD45+CD4+ T cells percent in total viable cells assessed by flow cytometry in mice from each group on day 4 (**F**), day 8 (**G**) and day14 (**H**) after the treatment.

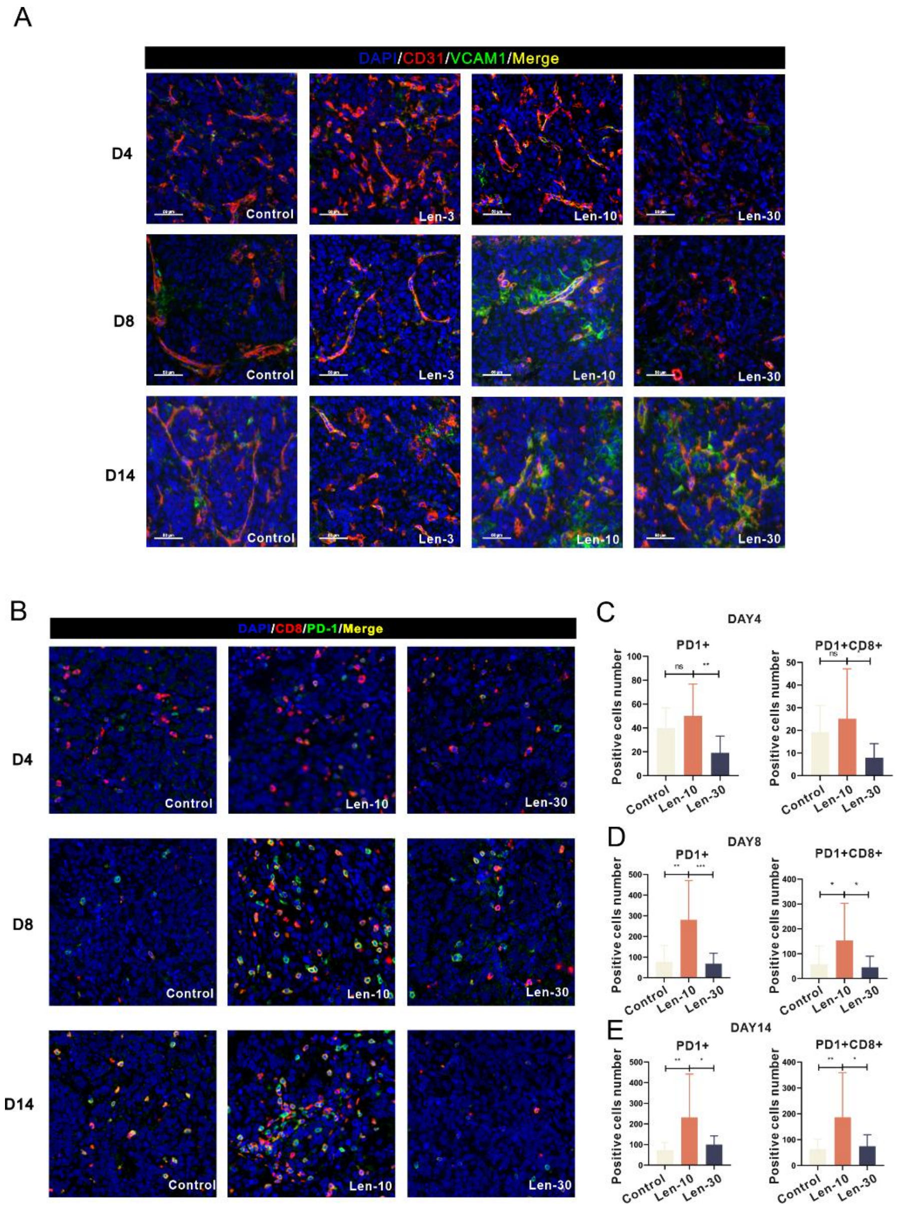

Fig. S6 (**A**) Representative multi-plex IHC staining images of VCAM1 (green) and CD31 (red) in mice from each group on day 4, 8 and 14 after the treatment. (**B**) Representative multi-plex IHC staining images of PD-1 (green) and CD8 (red) in mice from each group on day 4, 8 and 14 after the treatment. (**C-E**) Statistical graphs of PD-1+ cell number and PD-1+CD8+ T cell number on day 4 (**C**), day 8 (**D**) and day 14 (**E**) after the treatment.

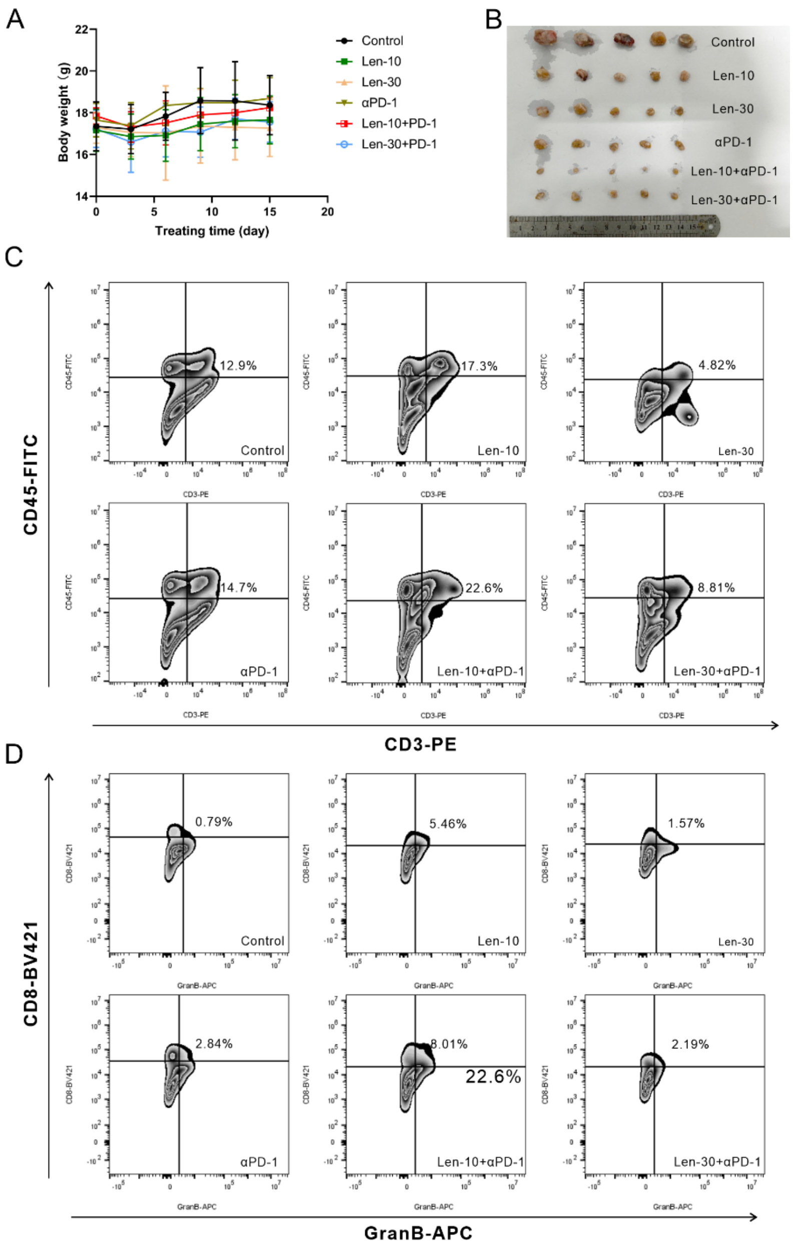

Fig. S7 (**A**) Body weight changes of the subcutaneously implanted tumors in mice from each group beginning at the day of tumor inoculation in the combined therapy (n = 5 per group). (**B**) Tumors isolated from mice of each treatment group 15 days after tumor inoculation. (**C-D**) Representative flow cytometry analysis of the CD45+CD3+ T cells (**C**) and CD8+GranBGzmb+ T cells (**D**) in each group on day 15 after the treatment.

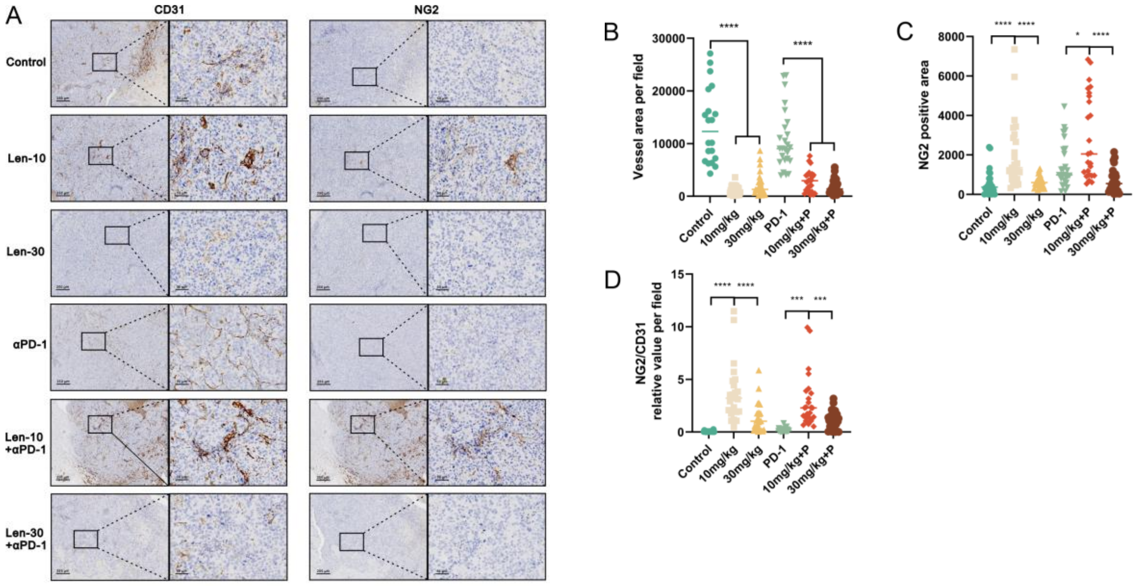

Fig. S8 (**A**) Representative IHC staining images of CD31 and NG2 in mice from each group. (**B-D**) Statistical graphs of CD31 positive area (**B**), NG2 positive area and NG2/CD31 relative area value S (**D**) in mice from each group assessed by IHC staining.

Supporting Video (shown in supporting files)

Video. S1 The dynamic perfusion video in the control group (**A**), Len-3 group (**B**), Len-10 group (**C**) and Len-30 group (**D**) in the immunodeficient mouse model on day 8 after the treatment.

Video. S2 The dynamic perfusion video in the control group (**A**), Len-3 group (**B**), Len-10 group (**C**) and Len-30 group (**D**) in the immunocompetent C57BL/6 mouse model on day 8 after the treatment.

Supplementary Materials and Methods

***In vivo* Experiments**

The NCG mice were subcutaneously injected with a total of 2×10^6 PLC/PRF/5 cells suspended in 100 μL of phosphate-buffered saline (PBS) containing 50% Matrigel basement membrane matrix (BD Biosciences, San Jose, CA, USA) and then randomly assigned to 4 groups of 5 mice per group. The mice in the control group were treated with a 0.5% (w/v) carboxymethyl cellulose–Na solution (CMC; Selleck Chemicals LLC, Houston, TX, USA). The mice in the other three groups were orally treated with 3 mg/kg/day (Len-3), 10 mg/kg/day (Len-10) and 30 mg/kg/day (Len-30) lenvatinib, respectively, at one week after inoculation. Tumor volume and body weight were measured every 3 days, and tumor growth was calculated using the following formula: $0.5 \times$ larger diameter $\times$ (small diameter)^2. Animal body weight was monitored as an indicator of treatment-related systemic toxicity. All mice were euthanized and executed at 16 days after inoculation and the tumors were harvested, photographed, weighed and then embedded in paraffin for subsequent analysis.

To evaluate the effect of lenvatinib on tumor vasculature in an immunocompetent microenvironment. The C57BL/6 mice were subcutaneously injected with a total of 1×10^6 Hep1-6 cells suspended in 100 μL of PBS containing 50% Matrigel basement membrane matrix and randomly assigned to the 4 groups (Control, Len-3, Len-10 and Len-30) of 15 mice per group. The method of lenvatinib administration was the same as above. Tumor growth was also measured and calculated. All mice were euthanized and executed the indicated timepoints (day 4, day 8 and day14) after the beginning of the treatments and the tumors were harvested for subsequent analysis.

For the combined therapy of lenvatinib and anti-PD-1 antibody, the tumor-bearing C57BL/6 mice were randomly assigned to 6 groups of 5 mice per group. Lenvatinib treatment at various doses was initiated 7 days after tumor cell inoculation and administered daily by oral gavage. Anti-mouse PD-1 CD279) antibody (BE0146; BioXcell, Lebanon, NH, USA) was initiated at the same time but administered at 200 mg/mouse by intraperitoneal injection every 3 days. Tumors were monitored once every 5 days using an *in vivo* IVIS Lumina Series III imaging system (PerkinElmer, Walthan,

MA, USA) for 15 days. At the end of the treatment, tumor-bearing mice were anesthetized and tissues were harvested for analysis and measurement.
